# Sex separation unveils the functional plasticity of the vomeronasal organ in rabbits

**DOI:** 10.3389/fnmol.2022.1034254

**Published:** 2022-10-21

**Authors:** Paula R. Villamayor, Julián Gullón, Luis Quintela, Pablo Sánchez-Quinteiro, Paulino Martínez, Diego Robledo

**Affiliations:** ^1^Departamento de Zooloxía, Xenética e Antropoloxía Física, Facultade de Veterinaria, Universidade de Santiago de Compostela, Lugo, Spain; ^2^Departamento de Anatomía, Producción Animal e Ciencias Clínicas Veterinarias, Facultade de Veterinaria, Universidade de Santiago de Compostela, Lugo, Spain; ^3^COGAL SL, Cuniculture Company, Rodeiro, Spain; ^4^Departamento de Patoloxía Animal, Facultade de Veterinaria Universidade de Santiago de Compostela, Lugo, Spain; ^5^The Roslin Institute, The Royal (Dick) School of Veterinary Studies, The University of Edinburgh, Edinburgh, United Kingdom

**Keywords:** vomeronasal organ plasticity, sex separation, socio-environmental conditions, rabbit chemocommunication, RNAseq, pheromones, reproduction

## Abstract

Chemosensory cues are vital for social and sexual behaviours and are primarily detected and processed by the vomeronasal system (VNS), whose plastic capacity has been investigated in mice. However, studying chemosensory plasticity outside of laboratory conditions may give a more realistic picture of how the VNS adapts to a changing environment. Rabbits are a well-described model of chemocommunication since the discovery of the rabbit mammary pheromone and their vomeronasal organ (VNO) transcriptome was recently characterised, a first step to further study plasticity-mediated transcriptional changes. In this study, we assessed the plastic capacity of the rabbit male and female VNO under sex-separation vs. sex-combined scenarios, including adults and juveniles, to determine whether the rabbit VNO is plastic and, if so, whether such plasticity is already established at early stages of life. First, we characterised the number of differentially expressed genes (DEGs) between the VNO of rabbit male and female under sex-separation and compared it to sex-combined individuals, both in adults and juveniles, finding that differences between male and female were larger in a sex-separated scenario. Secondly, we analysed the number of DEGs between sex-separated and sex-combined scenarios, both in males and females. In adults, both sexes showed a high number of DEGs while in juveniles only females showed differences. Additionally, the vomeronasal receptor genes were strikingly downregulated in sex-separated adult females, whereas in juveniles upregulation was shown for the same condition, suggesting a role of VRs in puberty onset. Finally, we described the environment-modulated plastic capacity of genes involved in reproduction, immunity and VNO functional activity, including G-protein coupled receptors. Our results show that sex-separation induces sex- and stage-specific gene expression differences in the VNO of male and female rabbit, both in adults and juveniles. These results bring out for the first time the plastic capacity of the rabbit VNO, supporting its functional adaptation to specifically respond to a continuous changing environment. Finally, species-specific differences and individual variability should always be considered in VNO studies and overall chemocommunication research.

## Introduction

Chemosensory communication controls many vital social and sexual behaviours such as fighting or mating and strongly impacts individual behaviour. It demands accuracy and a dynamic range of stimuli perception as well as highly sensitive transduction mechanisms. In mammals, chemical stimuli are predominantly detected through the vomeronasal system (VNS), considered as a hardwired system in charge of detecting innate and stereotyped chemosignals (Oboti and Peretto, 2014). However, since a few decades ago, investigations have been pointing toward a more dynamic VNS system. For instance, its high degree of plasticity was described by Ichikawa (1998), especially regarding synaptic morphology variation in the accessory olfactory bulb, the first centre for vomeronasal signal integration within the central nervous system. Lately, the VNS has entered a “new era” in which it was finally recognised as a system with capacity for adaptative learning, and experience- and state-dependent plasticity (Lanuza et al., 2014; Mohrhardt et al., 2018; Marom et al., 2019; Trouillet et al., 2021; Villafranca-Faus et al., 2021).

Chemical stimuli are released by one individual to the external world and detected by conspecifics, predominantly by vomeronasal receptors (VRs) located in vomeronasal sensory neurons (VSNs) within the vomeronasal organ (VNO) (Francia et al., 2014). Vomeronasal receptors are highly fine-tuned to be able to respond efficiently to continuous environmental changes. The two main types of vomeronasal receptors, vomeronasal receptors type 1 (V1R) and vomeronasal receptors type 2 (V2R), have been involved in the detection of physiological status of conspecifics by binding distinct classes of sexual steroids (V1R), and in sex-related behaviours (V2R), such as puberty acceleration, female attraction or male aggressiveness (Tirindelli, 2021). Additionally, in mice, a multigene family of non-classical class I major histocompatibility complex (MHC) genes (H2-Mv), were found co-expressed with V2R (Ishii et al., 2003) and, even though their functionality has not been fully explored, they contribute to ultrasensitive chemodetection by a subset of VSNs (Leinders-Zufall et al., 2014). Another type of vomeronasal receptors, representing an expansion of the family formyl peptide receptors (FPRs), has been identified in vomeronasal neurons in mice (Liberles et al., 2009; Rivière et al., 2009). This family is also expressed in immune cells directly involved in innate immunity and in recognition of several inflammation-related molecules (Migeotte et al., 2006; Bufe et al., 2012; Dietschi et al., 2017; Weiß and Kretschmer, 2018). Therefore, the VNS appears to be closely related to the immune system and its role in pathogen sensing (Bufe et al., 2019), detection and avoidance of sick conspecifics (Boillat et al., 2015) and genetic compatibility (Chamero et al., 2012), has been previously established. Due to the wide diversity of species-specific chemical stimuli perceived by the VNO, it is likely that other unknown types of vomeronasal receptors might be found in the VNO. For instance, it has been reported that VSNs are highly sensitive to excreted sex-steroids, glucocorticoids and bile acids (Doyle and Meeks, 2018) although their plasma membrane receptors remain unclear. VNO cytoplasmatic sex-steroid receptors may arise as potential candidates to perceive the physiological state of a given individual.

The VNO also shows a high plasticity range to cope with environmental changes. Marom et al. (2019) have recently determined that responses triggered by VSNs are highly plastic and greatly vary upon experience. Also, it is well-established that VSNs undergo regeneration throughout life both physiologically and after injury, thus supporting their plastic capacity (Martinez-Marcos et al., 2005; Brann and Firestein, 2010). Importantly, experience-dependent plasticity triggers dramatic changes at the individual level in the abundance of specific functional types of VSNs, inducing specific behaviours (Xu et al., 2016). Such individual variability adds further complexity to the study of the VNO. Experience has been documented to affect gene expression in VSNs, and, in particular, to that of vomeronasal receptors (VRs), but its correlation to neuron abundance has not been determined (Zhang et al., 2010; Broad and Keverne, 2012). Recently, long-term (6 months) exposure to a particular social environment (sex-separation conditions) has proved to induce changes in the gene expression of mice VNO both in the abundance (*in situ* Hybridization) and gene expression [RNA sequencing (RNAseq)] of VSNs and VRs (Santoro and Jakob, 2018; Van der Linden et al., 2018). However, it remains unclear whether shorter exposure to this particular condition would follow a similar plasticity. Also, since gene expression points to the function and pathways of specific genes, we argue that transcriptional changes might be the main mechanism by which olfactory sensory systems adapt to a particular environmental change. Indeed, despite only few studies have approached transcriptional changes in the VNO, data from the main olfactory epithelium (MOE) strongly supports transcriptional changes underlying sensory adaptation (Tsukahara et al., 2021). Recently, it has been determined that olfactory sensory neurons adapt to a particular environment via the activation of highly specific transcriptomic programmes (Flores-Horgue et al., 2022). Since the VNO is another sensorial structure which needs to adapt and react to particular scenarios, it seems reasonable that the VNO follows similar mechanisms of transcriptional adaptation. This does not exclude the potential existence of other adaptive mechanisms based on changes in neuron abundance, specifically of VSNs expressing VRs, but to our knowledge no data support this hypothesis.

Mice have been the gold standard species in chemocommunication studies. However, the species-specific nature of the VNS makes it necessary to broaden the species range to achieve reliable conclusions. Rabbits are considered a suitable model of chemocommunication since the discovery of the rabbit mammary pheromone (Schaal et al., 2003). A previous VNO anatomical and transcriptomic study on this species demonstrated its complex nature (Villamayor et al., 2018, 2021). Specifically, Villamayor et al. (2021) updated the number of VRs (128 V1Rs and 67 V2Rs) and showed species-specific expansions of ancestral genes in the rabbit VR repertoire compared to mice, which are generally distributed in a few rabbit-specific clades. They also showed expression of a wide range of VRs and other genes involved in communication and reproduction (Villamayor et al., 2021). In this study, we targetted for the first time the potential plasticity of the rabbit VNO. We used RNAseq to study the gene expression of the rabbit VNO under sex-separation and sex-combined conditions in adults (6 months old), considered as a long-term exposure and in which neural turnover is expected, and juveniles (40 days old), to decipher whether sensory plasticity starts at earlier stages. We found VNO plastic changes underlying sex-separation in male and female adults. Surprisingly, in juveniles, VNO plasticity was found in females but not in males, a feature that points to a potential relationship between the VNO activity and the onset of puberty. Special attention was focussed on the VR repertoire, where we found striking downregulation of VR genes in adult females when sex-separated. Other features such as expression changes of sex-steroid receptors or specific G protein coupled receptors were also detected. All in all, transcriptional modulation appears to play an important role in sensory adaptation, suggesting that the VNO may evolve to respond to the environment via transcription modulation since early life stages.

## Materials and methods

### Experimental design

**FIGURE 1:**
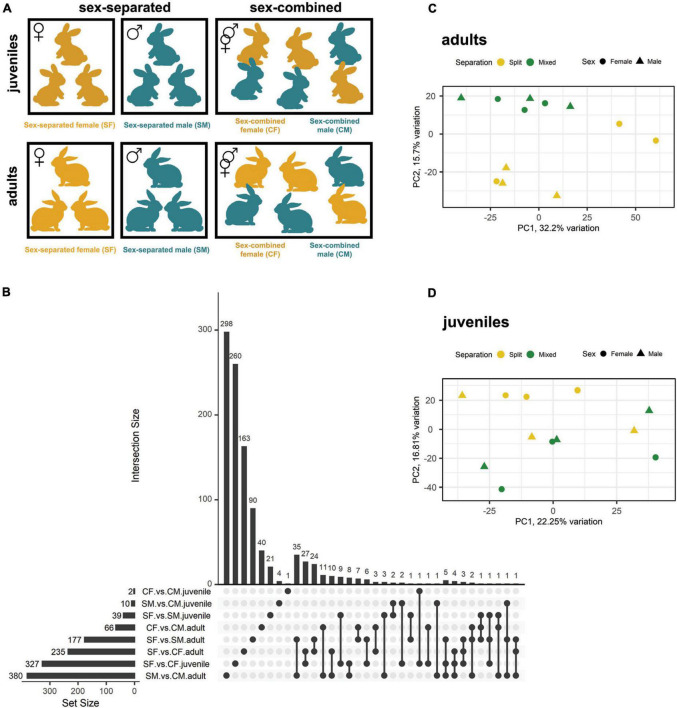
Gene expression differences between male and female rabbits in VNO tissue, both in adults and juveniles, under different socio-environmental conditions. **(A)** Experimental design. From day 0 until day 40 [first sampling point (juveniles)] and day 180 [second sampling point (adults)], rabbits experienced either a sex-separated environment or a sex-combined environment. **(B)** Upsetplot showing the number of DEGs of each experimental group and those shared between groups. The bars representing the intersection size show the number of DEGs shared by the experimental groups highlighted by black dots in the matrix panel below. **(C,D)** Principal component analysis (PCA) where sex-separated and sex-combined males and females are plotted, both in panel **(C)** adults and panel **(D)** juveniles.

We employed (1) male and female rabbits that were separated at birth (sex-separated) so that males and females did not have contact with members of the opposite sex, and compared their VNO transcriptome to (2) male and female rabbits that were reared together since birth (sex-combined). Two experimental time points [juveniles (40 days) and adults (6 months)] were considered. The two sampling points were selected following Van der Linden et al. (2018); according to these authors, 6 months would ensure enough time for experience-dependent changes in the abundance of sensory neurons subtypes. Further, we added juvenile individuals to ascertain whether these changes are already occurring at earlier stages. Separated female (SF), separated male (SM), combined female (CF), and combined male (CM) groups were constituted both for juvenile and adult sampling (Figure 1A; Supplementary Figure 1). To this end, all animals were reared under the same farm environment and management protocols.

On day 0, kits were re-distributed among mothers, so that each mother took care of eight kits coming from different mothers. For the sex-separated condition, female and male kits were separated since birth in different litters. We employed 50 litters/condition (50 for sex-separated females and 50 for sex-separated males) to ensure that animals were surrounded by individuals under the same condition, but only three individuals per condition/sex/stage were analysed for gene expression.

A detailed scheme of the organisation of animals per rooms can be found in Supplementary Figure 2. Briefly, four different rooms were employed in the experiment: room a, c, d for sex-separated conditions, and room b for sex-combined condition. All rooms had three double rows with capacity for 280 mothers with their litters per row (a total of 840 mothers/room) (Supplementary Figure 2). For the sex-separation condition, we first employed room a, in which male and female litters were placed in different rows in opposite sides of the room to avoid any type of contact between sexes. Weaning took place on day 30, mothers were then taken out of the boxes while kits remained until day 40 (sampling point 1: juveniles). 60 males and 60 females of 40 days old were then separated into different rooms (room c and d for males and females, respectively) of the farm. We kept four animals/cage and used 15 cages (60 rabbit males in room c and 60 rabbit females in room d), to ensure the same environment until 6 months old (sampling point 2: adults). For the sex-combined group we used a different room, room b, in which four kits of each sex were cared per mum (eight in total). We arranged 100 mothers, each with their own litter to ensure exposure to the same environment. The protocol was similar as for the sex-separated scenario, but no changes between different rooms were performed. Of note, since males and females were kept together to ensure direct contact between individuals, kits born during the 6 months of the experiment were removed from the box on day 0 to avoid exposure to pup-specific odours [same criteria as Van der Linden et al. (2018)]. Standard farm routines were followed, such as litter rearrangement to ensure all kits had similar weight, but always according to the experimental criteria. No signs of aggression were detected in any of the experimental conditions tested, likely due to continuous cohabitation since birth (Liberles, 2014).

### Sampling

Twenty-four animals, separated in 12 juveniles and 12 adults of 40 and 180 days, respectively, were used including the same number of males and females within each group. All animals pertained to a commercial hybrid (Hyplus strains PS19 and PS40 for female and male, respectively) and were maintained on a farm (COGAL SL, Rodeiro, Spain) under the same temperature conditions (18–24°C), dark-light cycles of 12:12 h and *ad libitum* feeding and drinking. All individuals were humanely sacrificed by an abattoir of the same company, following strict ethical farm conditions and in accordance with the current legislation. Sex-separated animals were sacrificed at the beginning of the day to avoid any type of contact with members of the opposite sex, and males and females were sacrificed in consecutive different days for the same reason. Their heads were separated from the carcasses in the slaughtering line and the whole double VNO structure was immediately dissected out after opening the lateral walls of the nasal cavity and removing the palate and nasal turbinates. Samples were immersed in Trizol and kept in ice (∼4°C). Tissue was homogenised at the sampling point using a mixer to guarantee the whole tissue sample was soaked by Trizol–due to the double bone and cartilage envelope of the rabbit VNO. After 20 min, all samples were stored at −80°C for further RNA extraction.

### Transcriptomic analysis

RNA extraction, library preparation and bioinformatic analysis was previously described in Villamayor et al. (2021). Briefly, libraries were sequenced on an Illumina NovaSeq 6000 150 bp PE run by Novogene. The quality assessment and the filtering and removal of residual adaptor sequences were performed using FastQC v.0.11.7^1^ and on Trimmomatic v.0.39 (Bolger et al., 2014), respectively. Filtered reads were aligned against the rabbit genome (OryCun2.0; Carneiro et al., 2014) and assigned to genes based on the latest annotation of the rabbit genome (Yates et al., 2020) using STAR v.2.7.0e (Dobin et al., 2013) two-pass mode.

Gene count data were used to calculate gene expression and estimate differential expression (DE) using the Bioconductor package DESeq2 v.1.28.1 (Love et al., 2014) in R v.3.6.2 (R Core Team, 2017). Size factors were calculated for each sample using the “median of ratios” method and count data were normalised to account for differences in library depth. Normalised reads were used as a measure of expression and were calculated by taking the average of the normalised count values, dividing by size factors, taken over all samples. This corresponds to the “basemean” obtained with DESeq2. Next, gene-wise dispersion estimates were fitted to the mean intensity using a parametric model and reduced toward the expected dispersion values. Finally, differential gene expression was evaluated using a negative binomial model that was fitted for each gene, and the significance of the coefficients was assessed using the Wald test. The Benjamini-Hochberg false discovery rate (FDR) correction for multiple tests was applied, and transcripts with FDR < 0.05 were considered differentially expressed genes (DEGs). Hierarchical clustering and principal component analysis (PCA) were performed to assess the clustering of the samples and identify potential outliers over the general gene expression background. The R packages “pheatmap,” “PCAtools,” “EnhancedVolcano,” and “UpSetR” were used to plot heatmaps, PCA, volcano plots, and the upsetplot, respectively. Gene functional annotation was performed by searching all rabbit VNO gene ontology (GO) terms with PANNZER2 (Törönen et al., 2018) and then assessing the enriched terms for each comparison of our study (SF vs. SM, CF vs. CM, SF vs. CF, SM vs. CM) and also for each condition independently (SF, SM, CF, and CM) both in juveniles and adults, by using AgriGO v2.0 (Tian et al., 2017). Gene BLAST analyses were performed using Ensembl BLASTN tool.^2^

In parallel, we also performed our analysis using kallisto (Bray et al., 2016), which pseudoaligns reads to a reference, producing a list of transcripts that are compatible with each read while avoiding alignment of individual bases, which is what STAR does. Similar results were obtained with both software, STAR and kallisto, and therefore STAR was used by default in our analysis.

## Results

The main goal of this study was to analyse the plasticity and capacity for sensorial adaptation of the VNO in response to diverse socio-environmental conditions. We characterised gene expression in the VNO for the four conditions (SF, SM, CF, and CM) and two stages (adults and juveniles) using RNAseq.

### Sex-separation induces sex-specific differences in gene expression of male and female rabbits

We first identified DEGs (FDR < 0.05) between females and males under sex-separated (SF vs. SM) or sex-combined conditions (CF vs. CM) in the rabbit VNO of adults and juveniles (Table 1; Supplementary Data 1). We found 177 and 66 DEGs, when comparing adults in sex-separated and sex-combined conditions, respectively. The same comparison in juveniles rendered significantly fewer DEGs, but following a similar trend as in adults: 39 DEGs when sexes were separated, while only 2 when sexes were combined (Table 1; Figure 1B). These data show that sex-separation determines greater transcriptomic changes between males and females in the rabbit VNO than in the sex-combined scenario, not only in adults but also in juveniles (Figures 1C,D; Supplementary Figure 3). Very few DEGs were shared between both scenarios (sex-separated and sex-combined) in adults (10 genes; Figure 1B; Supplementary Data 2), demonstrating that the vast majority of DEGs were condition-specific. Seven of those 10 genes were upregulated in males in both scenarios and included genes such as cholecystokinin (CCK), major allergen I polypeptide chain 1 (FEL1A) or excitatory amino acid transporter 3 (SLC1A1). The latter is a glutamate transporter gene which was previously involved in fear response behaviour, and also obsessive–compulsive disorder (Shugart et al., 2009) and fertility (Liu et al., 2017) in males.

**TABLE 1:** Total differentially expressed genes among the specific experimental conditions in the VNO of adult and juvenile rabbits and its comparison with previous data in mice VNO (Van der Linden et al., 2018).

Comparisons	Number of DEGs (adult)	Number of DEGs (juvenile)	Number of DEGs (adult mice) (Van der Linden et al., 2018)
SF vs. SM	177	39	106
CF vs. CM	66	2	64
SF vs. CF	235	327	64
SM vs. CM	380	10	35

SF, separated females; SM, separated males; CF, combined females; CM, combined males.

In juveniles, all the 39 DEGs found were specific of the sex-separated scenario, suggesting that sex-separation induces much greater differences between sexes at this stage than sex-combined. Only 3 of these DEGs were also detected in the same comparison in adults (Figure 1B; Supplementary Data 2), therefore, there is almost no overlap between stages. These results suggest that juveniles and adults have sex-specific gene expression repertoires and that both stages should be analysed independently.

All in all, results indicate that sex-separation induces VNO gene expression differences between males and females in the adult and juvenile rabbit VNO. Adult rabbits exhibit the largest divergence in the number of genes expressed (111 genes) and therefore, a higher plasticity under the two environmental scenarios.

### Vomeronasal organ transcriptome changes depending on environmental conditions in a sex-specific manner

The differential gene expression detected between females and males subjected to different environmental conditions (sex-separated vs. sex-combined) could arise via changes in one sex or in both sexes. To decipher the influence of each sex, we compared DEGs between sex-separated and sex-combined females (SF vs. CF) and males (SM vs. CM), both in adults and juveniles (DEGs combined vs. separated). In adults, 235 (SF vs. CF) and 380 (SM vs. CM) DEGs were detected, while in juveniles the difference was much higher in females (327; SF vs. CF) than in males (10; SM vs. CM) (Figure 1B; Supplementary Figure 4; Supplementary Data 3). It may be speculated that the big transcriptomic changes in the VNO of female juveniles could play a role on the onset of female puberty. Moreover, DEGs were mostly sex-specific for each condition, and in adults only 15 DEGs were shared between male and female comparisons (Figure 1B; Supplementary Data 4). Remarkably, among these 15 DEGs we found the G-protein GPA1, which is known to mediate mating pheromone signal transduction in yeast and is found expressed in V1R and V2R expressing neurons in mammals (Alvaro and Thorner, 2016). The fold change (FC) of this gene was among the highest in both sex comparisons (FC: −2.92 in females and −1.91 in males) being upregulated in the sex-separated condition for both sexes. In juveniles, only two DEGs overlapped between sexes out of 10 DEGs detected in males (Figure 1B; Supplementary Figure 4).

A higher overlap was observed when comparing the results of juveniles and adults of the same sex, especially in females, which shared 34 DEGs (Figure 1B; Supplementary Data 4). GPA1 was shared also in this case, highlighting the importance of this protein not only in adults but also at pre-puberal stages. In males, two genes overlapped between juveniles and adults among the 10 DEGs found in the juvenile comparison (Figure 1B; Supplementary Data 4).

These results indicate that adults of both sexes exhibit specific responses of their overall gene repertoires depending on the environmental conditions (sex-separated and sex-combined scenarios), which is also true for juvenile females. Therefore, when studying how environmental modulation affects VNO gene expression, variations in sex and stage are highly expected and should be carefully considered.

### Vomeronasal receptor repertoire undergoes significant downregulation in adult female vomeronasal organ under the sex-separation condition

We specifically analysed the gene expression of the vomeronasal receptor (VRs) repertoire considering its relevance for chemocommunication, especially on reproductive behaviour. We used a more conservative significance threshold of *p* < 0.01 (unadjusted) considering the low transcript abundance of VRs, as previously suggested (Van der Linden et al., 2018). We also considered as “suggestive” those genes with *p* < 0.05 (unadjusted) to broadly deepen into the VRs gene repertoire. First, we compared the number of VR DEGs between male and female under sex-separated or sex-combined conditions in adults and juveniles. In adults, we found four consistent (*p* < 0.01; 3 V1R and V2R) and 17 suggestive (*p* < 0.05; 12 V1R and 5 V2R) differentially expressed (DE) VRs in the sex-separated condition, all of them downregulated in females (Table 2; Supplementary Data 5). Conversely, only one suggestive DE VR (V1R) was found in the sex-combined condition. These results support the suppression of VRs expression in sex-separated females, while in the sex-combined scenario both sexes showed a similar VR gene expression profile. In juveniles, fewer DE VRs were detected, but their expression followed a similar pattern as in adults: 1 consistent (V1R) and 10 suggestive (3 V1R and 7 V2R) DEGs between females and males in the sex-separated condition and none in the sex-combined condition (Table 2; Supplementary Data 5). Contrary to adults, in juveniles all these 10 suggestive DE VRs were found upregulated in females (SF > SM; *p* < 0.05).

**TABLE 2:** Differentially expressed VRs among the specific experimental conditions in the VNO of adult and juvenile rabbits and its comparison with previous data in mice VNO (Van der Linden et al., 2018).

Comparisons	Number of DEGs (adult)	Number of DEGs (juvenile)	Number of DEGs (adult mice) (Van der Linden et al., 2018)
SF vs. SM	4 (17)	1 (10)	12
CF vs. CM	0 (1)	0	10
SF vs. CF	3 (12)	9 (27)	0
SM vs. CM	2 (8)	0 (10)	4

*p* < 0.01, consistent difference; in brackets *p* < 0.05, suggestive difference; (both unadjusted). SF, separated females; SM, separated males; CF, combined females; CM, combined males.

We then aimed to evaluate whether the DE VR repertoire is shared between adults and juveniles. We found no overlapping VRs in the four comparisons (SF vs. SM; CF vs. CM in both juveniles and adults; *p* < 0.05), underscoring that the DE VRs are highly sex- and stage-specific, and that the environment critically influences VRs expression. Remarkably, these results contrast with those found by Van der Linden et al. (2018) in the mice VNO, where five DE VRs that overlapped between (1) male and female sex-separated (SF vs. SM) and (2) male and female sex-combined (CF vs. CM) mice were detected. Accordingly, they concluded that the environment has little effect on VRs gene expression. Taken together, these results indicate that each species could show its own VR expression pattern, depending on socio-environmental conditions, thus reflecting how dynamic and plastic the VNO must be for external stimuli perception.

We then characterised whether DE VRs detected between female and male rabbit subjected to environment conditions (sex-separated vs. sex-combined) could originate from changes in one sex or in both sexes. In female adults, we found three consistent (V1R, V2R) and 12 suggestive (eight V1R and four V2R) DE VRs between sex-separated and sex-combined individuals (SF vs. CF), whereas in male adults, we detected two consistent (one V1R and one V2R) and eight suggestive (three V1R and five V2R) DE VRs for the same comparison (SM vs. CM) (Table 2; Supplementary Data 6). So, adults of both sexes undergo changes in the expression of VR genes depending on the environmental conditions. Remarkably, most VRs were upregulated in the sex-combined condition (CF > SF; CM > SM), which indicates that sex-separation does not promote VRs expression in the adult rabbit VNO, especially in females. Additionally, there was no overlap between sex comparisons (SF vs. CF and SM vs. CM), suggesting sex-specific gene expression repertoires under environmental modulation. In juveniles, we found nine consistent (one V1R and eight V2R) and 27 suggestive (6 V1R and 21 V2R) DE VRs between sex-separated and sex-combined females (SF vs. CF), whereas we detected no consistent and 10 suggestive (five V1R and five V2R) DE VRs for the same comparison in males (SM vs. CM) (Table 2; Supplementary Data 6). Contrary to adults, in juveniles all VRs were upregulated in sex-separated animals both in males and females. We found four genes overlapping between males and females for those comparisons (SF vs. CF and SM vs. CM), suggesting similar environmental influence on those genes for both sexes.

**FIGURE 2:**
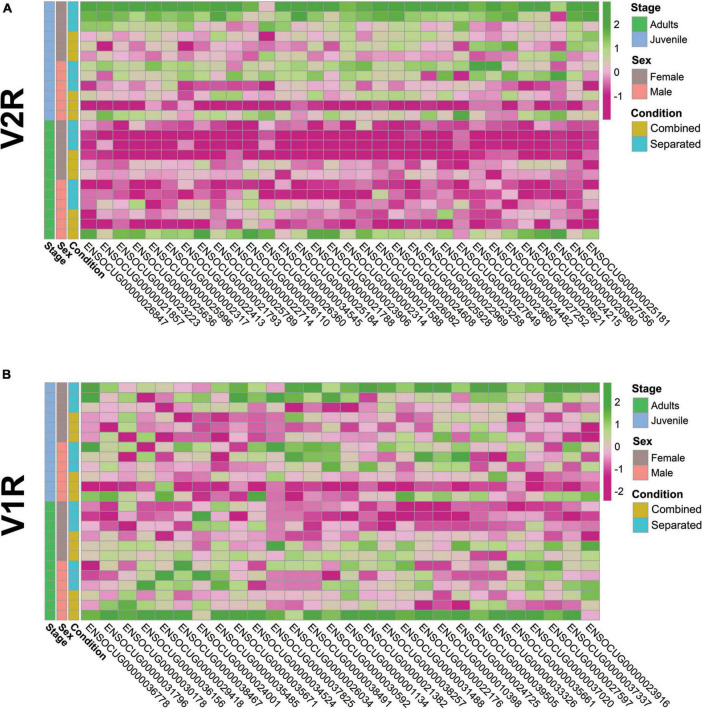
Heatmaps of differentially expressed **(A)** V2Rs (*p*-value < 0.05) and **(B)** V1Rs (*p* < 0.05).

Two main conclusions can be extracted from the VRs gene expression results: (1) rabbit VRs gene expression is sex- and stage-specific under different socio-environmental conditions (sex-separated vs. sex-combined) and (2) adult females in a sex-separated scenario showed the lowest VRs expression among all comparisons. For a more refined evaluation of VRs gene expression, we then visualised all V2R and V1R showing differential expression (consistent or suggestive) in any comparison (Figures 2A,B). V2Rs expression was significantly higher in juveniles than in adults regardless of sex and environment, and the lowest V2R expression was shown by sex-separated adult females. Interestingly, one sex-combined adult male and one sex-separated juvenile female showed specific upregulation of V2Rs compared to the rest of individuals (outliers), hampering significant conclusions due to the increased variation (Figure 2A). The pattern of V1R expression underwent greater interindividual variation than V2R, but still showed that sex-separated adult females display the lowest V1Rs expression (Figure 2B)–although not so extreme as for the V2R expression. Furthermore, the same two outlier individuals for V2Rs also showed higher overall V1Rs expression. The high interindividual variation raises the question of whether these two animals could be dominant individuals, being more prepared than others for mating or fighting purposes.

Individual variation of VRs expression has been previously described (He et al., 2008), but most VRs studies have emphasised results per group of individuals under the same experimental conditions (Van der Linden et al., 2018). Our data show significant intragroup variation in the VRs gene expression repertoire despite the similar conditions assessed (sex, stage and environment), highlighting the importance of individual variability that makes difficult extracting consistent conclusions between groups. Further VRs studies should always consider this interindividual variability trying to minimise error in the estimations, which means randomising genetic variation, homogenising as much as possible environmental conditions and increasing sample size.

### Environmental modulation triggers differential expression of vomeronasal organ genes involved in reproduction and sexual behaviour, supporting its plastic capacity to ensure individual survival

**FIGURE 3:**
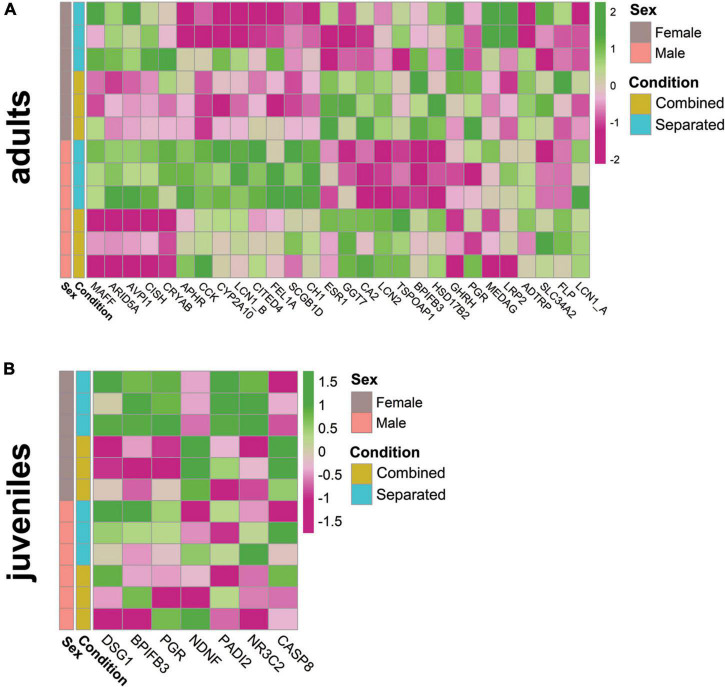
Heatmap of relevant reproduction-related genes in **(A)** adult male and female and **(B)** juvenile female rabbit VNO, comparing sex-separated vs. sex-combined conditions (SF vs. CF).

Adult males and females exhibited sex-specific expression differences of reproduction-related genes, subjected to environmental conditions. Genes belonging to the lipocalin family followed significant differential expression according to conditions in males and females (Figure 3A) [e.g., Lipocalin 1A (LCN1_A, ENSOCUG00000026763), SM > SF; lipocalin 1B (LCN1_B, ENSOCUG00000028189), SM > CM; lipocalin 2 (LCN2, ENSOCUG00000021002), CF > SF, CM > SM, SF > SM; and aphrodisin (APHR, ENSOCUG00000029236, SM > SF)], suggesting the specificity of different members of the lipocalin family. These genes may bind different putative sex-specific pheromones depending on the environmental conditions, which in turn would impact VNO pheromone-detection. We also found differential expression of BPIFB3 (ENSOCUG00000014883; CM > SM), a gene belonging to the BPI family that interacts with a major urinary protein (MUP10) in mice, a lipocalin binding pheromone protein affecting the sexual behaviour of females.^3^ A similar expression pattern (CM > SM) was found for the female-specific lacrimal gland protein (FLP, ENSOCUG00000027780), a gene previously identified as a lipocalin in hamsters which shows 85% protein sequence identity with male-specific submandibular salivary gland proteins (MSP) secreted in saliva and urine of male hamsters (Srikantan et al., 2005). Therefore, rabbit FLP may be other lipocalin subjected to environmental modulation, whose presence in the VNO points to a role as carrier of small putative hydrophobic pheromones. Further studies are required to determine the role of each lipocalin within the rabbit VNO. We also found differential expression of other important reproduction-related genes in adults, such as the sex-steroid binding progesterone receptor (PRG, ENSOCUG00000014693) and oestrogen receptor (ESR, ENSOCUG00000004829), both CF > SF, *p* = 0.057 (adjusted) and *p* = 0.014 (unadjusted), respectively. Other genes related to steroid-binding (FEL1A, CH1 CITED4, CISH, etc.) showed differential gene expression between conditions (Figure 3A; Supplementary Table 1). In juveniles, most of the analysed reproductive-related genes were found downregulated compared to adults (Supplementary Figure 5; Supplementary Table 1), probably because they have not reached puberty yet. Additionally, females but not males showed significant differences between sex-separated and sex-combined conditions (SF vs. CF) (Figure 3B). Reproductive-related genes, such as BPIFB3, DSG1 (desmoglein 1, which responds to progesterone) and PGR [this latter, *p* < 0.05 (unadjusted)], were differentially expressed between conditions (SF > CF) (Figure 3B; Supplementary Data 3), suggesting that gene expression changes of reproduction-related genes occur at least from day 40 in the female VNO. Interestingly, neuron derived neurotrophic factor (NDNF), (ENSOCUG00000002038), a gene directly related to GnRH neuron migration and the onset of puberty (Herbison, 2016; Messina et al., 2020; Oleari et al., 2021), was significantly upregulated in combined females compared to separated females (CF > SF) (Figure 3B; Supplementary Data 3), and therefore exposure to different environments may influence the timing of puberty. We previously showed that rabbit juveniles already expressed genes related to reproductive functions (Villamayor et al., 2021), and here we have proved that their expression is modulated by different environmental conditions.

Taken together, these results indicate that, in the adult and juvenile VNO, expression of several reproduction-related genes changes according to the environmental conditions (sex-separated, sex-combined) and some of them are sex-specific.

### Environmental changes modulate vomeronasal organ functional activity, thus adding complexity and flexibility to the vomeronasal organ sensory code

**FIGURE 4:**
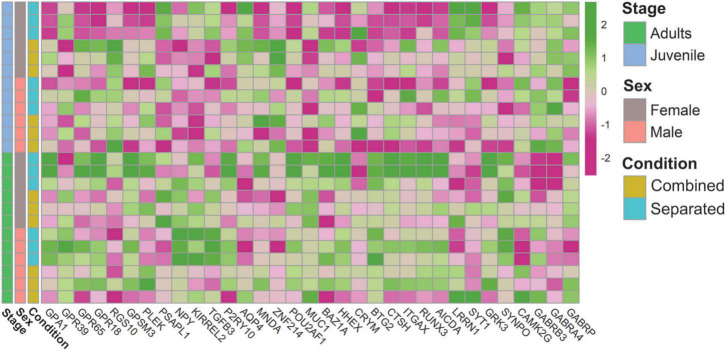
Heatmap of genes related to the VNO functional activity.

We next sought to determine whether the functional activity of the VNO varies among the experimental groups. Enrichment analyses revealed overrepresentation of G-protein related terms such as “G protein-coupled chemo attractant receptor activity” and “G protein-coupled receptor signalling pathway” among DEGs detected in sex-separated vs. sex-combined females (SF vs. CF), both in adults and juveniles (Supplementary Data 7D,E). In juveniles, these terms as well as others related to “response to stimulus” and “signal transduction” were directly linked to vomeronasal type-2 receptors (VN2R116) and the G protein GPA1 (Supplementary Data 7E). Both in juveniles and adults GPA1 was found upregulated in sex-separated females when compared to sex-combined females (SF > CF), and a similar pattern was found in adult males for the same comparison (SM > CM). Interestingly, a heatmap representation (Figure 4) shows that GPA1 is more expressed in adult separated females than in any other group. Considering that sensory transduction through the VNO proceeds via a G-protein-coupled mechanism (Berghard and Buck, 1996), our data showed that a high number of G-protein coupled receptor genes (GPR39, GPR65, GPR18), as well as genes which modulate (RGS10, GPSM3, PLEK) or inhibit (PSAPL1) the G-protein signalling activity, are differentially expressed among comparisons, especially between sex-separated vs. sex-combined female adults (SF vs. CF) (Supplementary Data 3; Figure 4). These results indicate that G-protein’s expression varies among comparisons, likely cooperating in the modulation of the vomeronasal signal transduction in response to specific environmental inputs. Another gene family that might be related to VNO signal transduction and worth mentioning is water channel aquaporins (AQPs). In the rabbit VNO transcriptome, we previously detected high expression of AQP2, AQP3, and AQP4 (Villamayor et al., 2021). Other studies proved the expression of AQP2 and AQP3 in mice olfactory organs but not in their sensory neurons (Ma et al., 2011), whereas AQP4 was the only aquaporine substantially expressed in VNO sensory neurons in rat (Ablimit et al., 2008). Remarkably, we detected AQP4 downregulated in the VNO of adult male rabbits when sex-separated compared to sex-combined (CM > SM) (Supplementary Data 3; Figure 4), thus suggesting that AQP4 undergoes gene expression changes in a sex-specific manner, yet their presence in VSNs in rabbits remains unknown. AQP4 might be involved in social behaviour and sexual preference by having a specific role in the VNO neuronal transduction. However, its physiological importance in sensory neurons of the VNO remains to be discovered (Ma et al., 2011).

Other enriched functions were detected under environmental changes (Supplementary Data 7). We found downregulation of biological processes GO terms such as “gene expression” and “regulation of transcription” in the list of DEGs between sex-separated females vs. sex-separated males (SF vs. SM) in adults (Supplementary Data 7B). In this comparison as well as in others such as sex-separated females vs. sex-combined females (SF vs. CF), both in juveniles and adults, we also found downregulation of terms related to “RNA metabolic processes” (Supplementary Data 7B,D,E). Among the genes involved in those functions, we found several transcription factors (MNDA, ZNF214, POU2AF1, MUC1), transcript repressors (BAZ1A, HHEX, CRYM, BTG2), modulators of gene expression (CTSH, ITGAX, positive regulation; and SERPINF1, negative regulation) and others related to epigenetic changes (RUNX3, AICDA). More specifically, genes involved in the regulation of synaptic transmission (LRRN1, SYT1, GRK3) as well as in synaptic plasticity (SYNPO, CAMK2G) and GABAergic activity (GABRB3 and GABRA4, GABRP) were also DE among comparisons (Supplementary Data 3; Figure 4), thus supporting VNO functional plasticity. All these genes might play a role in how sensory inputs are perceived by VNO (likely by modulating the expression of vomeronasal receptors) and how the electrical responses are sent to higher brain centres.

These results support that the VNO of male and female rabbits undergoes transcriptional changes according to environmental conditions, which might represent an adaptation of sensory responses to upcoming signals. These results are consistent with recent data by Flores-Horgue et al. (2022) in the MOE, in which odorant exposure led to reprogramming via the modulation of transcription.

### Vomeronasal organ as a first barrier in the detection of external stimuli, including harmful pathogens, and its relation with the immune system

The VNO is capable of detecting a broad range of chemical cues which fluctuate depending on given environmental scenarios. We found enriched molecular function GO terms related to “response to chemical/biotic stimuli and to other organisms” and “regulation of response to external stimulus,” especially in sex-separated vs. sex-combined females (SF vs. CF), both in juveniles and adults, but also in sex-separated females vs. sex-separated males (SF vs. SM) in adults (Supplementary Data 7B,D,E). The VNO is provided with different types of vomeronasal receptors to detect those stimuli. In addition to V1R and V2R receptors, several H2-Mv (belonging to the class I MHC family) and FPRs (linked to pathogen sensing) genes have been identified in mice (Ishii et al., 2003; Liberles et al., 2009; Rivière et al., 2009). We blasted the nine mice H2-Mv receptors against the rabbit VNO transcriptome (Villamayor et al., 2021) and found significant hits with six genes belonging to the class I MHC in rabbits. Among them, two showed gene expression variation depending on sex-combined vs. sex-separated scenarios (Supplementary Data 8). These results suggest that different vomeronasal receptors belonging to the MHC class I receptors previously unidentified in rabbits might play a role as vomeronasal receptors with plastic capacity to sensitively detect specific external cues in the rabbit VNO.

**FIGURE 5:**
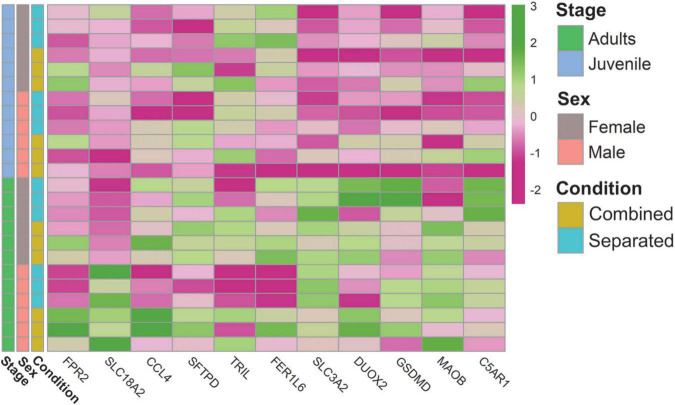
Heatmap of genes related to the VNO capability of detecting external stimuli, harmful pathogens and its relation with the immune system.

The VNO has also been involved in the detection of harmful bacteria and sick conspecifics, likely through FPRs (Bufe et al., 2019). The rabbit genome does not contain expansion of VNO FPRs, but the rabbit VNO transcriptome includes the immune receptors FPR2 and FPR1 (Villamayor et al., 2021). Here we found upregulation of FPR2 and FPR1 in sex-combined individuals, especially in males. Specifically, FPR2 (ENSOCUG00000008606) was upregulated in sex-combined adult males, compared to sex-separated adult males (CM > SM). FPR1 (ENSOCUG00000008600) was upregulated in sex-combined males and also in sex-combined females, compared to their separated counter partners [CM > SM; CF > SF, for both *p* < 0.01 (unadjusted)]. These results indicate that FPR2 and FPR1 are environment-modulated and therefore its immune function could be more complex than previously thought. Additionally, we found many molecular function GO terms related to “response to bacterium” and “defense response” involving DEGs of several comparisons (Supplementary Data 7). Specifically, some DEGs were related to response to toxic substances (SLC18A2, CCL4), defense response to bacteria (SFTPD, TRIL, FER1L6) and virus (SLC3A2, DUOX2), reaction to danger signals (GSDMD and MAOB), or showed specific defense response to Gramme-positive bacteria (C5AR1, also known to detect sensory perception of chemical stimulus). Other GO terms directly related to the immune system such as “antigen binding,” “immune response,” “MHC protein complex” were also found among comparisons (Supplementary Data 7) as well as genes related to the immune system such as immunoglobulins, interleukines and chemokines (Figure 5; Supplementary Data 3).

Overall, these results indicate that the VNO plays an important plastic role at detecting chemical stimuli and harmful bacteria, in narrow connection with the immune system, thus acting as an interface between the external world and the central nervous system for modulating responses against potential dangers. However, despite the clear importance of the VNO in the detection of harmful stimuli, we are still far from having a clear picture of species-specific VNO receptor repertoire in charge of mediating these fundamental behaviours.

## Discussion

This discussion addresses important key points of our results, integrating them in a broad context of VNO chemoperception. First, a comparison between our results and those found in mice (especially regarding VRs) confirmed that VNO plasticity, via transcriptional modulation, follows a different rationale according to species. Then, we discussed the potential role in pheromone perception of two important gene families–lipocalins and sex-steroids–broadly expressed in the VNO. Finally, we argued that VNO activity, and in particular VRs expression, might be linked to puberty onset, a fundamental question which needs to be further explored. Altogether, our study may serve as a bottom line for future studies of VNO chemocommunication, in which specie-specificity as well as VNO plasticity should always be considered.

### Vomeronasal organ and vomeronasal receptor repertoires show species-specific and environmentally modulated expression: a comparison between rabbits and mice

Our results proved that sex-separation induces sex-specific differences in the rabbit VNO gene expression, and this is consistent with previous results in adult mice (Van der Linden et al., 2018). However, some features are species specific. When comparing sex-separated to sex-combined individuals, the VNO of juvenile rabbits followed a similar expression pattern to that of adult mice [a higher number of DEGs between sex-separated and sex-combined females than between the same comparison in males (SF vs. CF > SM vs. CM)]. This difference was more exacerbated in juvenile rabbits than in adult mice, to the extent in which juvenile male rabbits showed almost no DEGs under the different environmental conditions, whereas females showed extreme variation. On the other hand, the VNO of adult rabbits follows an opposite rationale [higher number of DEGs between sex-separated and sex-combined males than between the same comparison in females (SM vs. CM > SF vs. CF)]. In this case, both sexes underwent important VNO transcriptomic changes.

Despite males and females exhibit striking sexually dimorphic behavioural responses to pheromones, and VNO shows sexually dimorphic anatomical features (Guillamón and Segovia, 1997), its VRs repertoire has proved to be very similar in both sexes in mice (Ibarra-Soria et al., 2014; Duyck et al., 2017). Interestingly, our results showed differences in the expression pattern of VNO and VRs between males and females when they were exposed to specific socio-environment conditions. Additionally, we tested sex specificity, meaning the extent to which DEGs found between sex-separated and sex-combined females overlap with those DEGs found in the same comparison in males. We hardly found DEGs overlapping between sexes in rabbits (6.38% in adults, 15 of 235 DEGs from SF vs. CF were also found in SM vs. CM; and 0.61% in juveniles, 2 of 327 DEGs from SF vs. CF were also found in SM vs. CM), in contrast with mice, which exhibited a much higher overlap between SF vs. CF and SM vs. CM (28.13%, 18 of 64 DEGs; see Figure 1G from Van der Linden et al. (2018). These data indicates that the VNO transcriptome is more sex-specific in rabbits than in mice when sex-separated and sex-combined conditions are tested. The fact that mice were laboratory domesticated animals, while rabbits were under farm conditions, might be connected to this observation, e.g., reduced plasticity in laboratory animals.

We then aimed at determining whether this sex-specific VNO expression is environmentally modulated. To do so, we compared the differential gene expression of the adult VNO between males and females considering both environmental conditions (sex-separated vs. sex-combined). We detected a rather small overlap between sex-separation and sex-combined scenarios (15.2% DEGs, 10 of 66 DEGs from CF vs. CM were also found in SF vs. SM). This result indicates that different environmental conditions give rise to different DEGs repertoires in the VNO of male and females rabbits, thus supporting the importance of environmental context in the overall VNO gene expression. In contrast, in the mice VNO, Van der Linden et al. (2018) found much higher overlap (43.8%; 28 of 64 DEGs) between the same comparisons [see Supplementary Figure 6A in Van der Linden et al. (2018)]. When studying the MOE, they found again an important overlap [39% (16 of 41 DEGs; see Figure 2A in Van der Linden et al. (2018)] between the same comparisons. However, authors argued little overlap in the adult mice MOE compared to a high overlap on the VNO despite both percentages (43.8 and 39%) are rather close, suggesting caution on this conclusion.

Considering that chemical stimuli detected through vomeronasal receptors may vary depending on a given environment, other important point was to decipher whether VRs expression in males and females is also environmentally modulated. By performing a similar comparison as above, we did not find overlap between sex-combined and sex-separation scenarios (CF vs. CM and SF vs. SM), both in adult and juvenile rabbits. In contrast, Van der Linden et al. (2018) found significant overlap between the two comparisons [50%, 5 of 10 VRs in CF vs. CM were also found in SF vs. SM; see Supplementary Figure 6E in Van der Linden et al. (2018)]. They argued that differences in the expression of VRs would be largely independent from socio-environment conditions and may instead be driven primarily by self-derived odours. However, the same comparison for olfactory receptors (ORs) found no overlap between comparisons (similar to what we found in rabbit VRs), concluding that the environment would be critical for sex-biassed ORs expression. Our data indicates that VRs expression repertoire in rabbits is environmentally modulated and the conclusions obtained by Van der Linden et al. (2018) in mice do not apply to rabbits. Therefore, the detection of both self- and opposite sex-derived odours in rabbits could be mediated by either VRs or ORs (this latter is matter of further investigation as to date it remains completely unexplored) depending on the perceived stimuli. Our results also highlight that generalised statements across species may lead to mistaken conclusions.

Additionally, Van der Linden et al. (2018) studied the expression of six VRs using RNA-FISH and only two of them showed consistent profiles with the RNAseq data. They argued that some VR expression differences observed by RNAseq may reflect differences in cellular VR transcript levels and not cell density. However, FISH might not be a suitable approach to characterise VRs plasticity under environment-modulated conditions, especially due to the high sequence similarity shared by different VRs. Instead, new–OMICS techniques such as single-cell and spatial transcriptomics could provide a more solid framework not only on VRs plasticity studies but also on a broader range of vomeronasal receptors and other VNO-related genes, to validate the correlation between transcript abundance and cell density depending on the environmental conditions.

All in all, we found little DEGs overlap in the rabbit VNO between males and females in both juveniles and adults, and in all scenarios tested. These results indicate that environmental modulation changes the VNO gene expression in a sex- and stage-specific manner in this species. This leads us to conclude that sex-separation is a condition which induces sex- and stage-VNO specific differences, and which is influenced by species (mouse vs. rabbit) tissues (vomeronasal vs. main olfactory organs) and individual variability. Our observations are consistent with the plastic capacity of the VNO to cope with different environmental scenarios, but further studies are needed to clarify the rationale behind this overwhelming gene expression heterogeneity.

### The lipocalin aphrodisin is upregulated in sex-separated adult male vomeronasal organ

Different members of the lipocalin family are involved in pheromone transport and can be found in a variety of body fluids such as urine (Pelosi and Knoll, 2022), tears (Stopkova et al., 2017), or saliva (Stopka et al., 2016). They can act either as carrier proteins for small hydrophobic like-pheromones [some of them expressed in the VNO (Miyawaki et al., 1994)] or directly as pheromones, such as darcin in mice (Roberts et al., 2010). APHR, a pheromone that belongs to the lipocalin family, is known to be released by hamster vaginal secretions (even before the onset of puberty) and parotid gland (Singer et al., 1986; Mägert et al., 1999) and it is detected by the male VNS eliciting copulatory behaviour in male hamster (Clancy et al., 1984; Kroner et al., 1996; Jang et al., 2001; Briand et al., 2004a). Different pheromone-like hydrophobic compounds were specifically bound onto natural APHR suggesting its role as pheromone transporter (Briand et al., 2004a,b).

We found for the first time expression of APHR in the VNO tissue of rabbits, being highly expressed in sex-separated adult males, compared to the same comparison in females (SM > SF). Therefore, its expression appears to be sex-specific and clearly depends on the environment. This could prepare separated males to be ready (receptors not saturated) to bind female-pheromones, eliciting copulatory behaviour in the presence of females. Also, it could selectively modulate the activation of vomeronasal receptors. Interestingly, when both sexes are reared together, the expression of this gene is lower (CM < SM), *p* = 0.013 (unadjusted), which could reflect saturation when males are continually exposed to the putative female pheromones. This would follow a similar rationale to the theory described for VRs “use it and lose it” (Van der Linden et al., 2018), which could then apply to other genes directly involved in pheromone-sensing. Of note, to date lipocalins were only found in the glandular region and vomeronasal mucosa of the VNO, but further studies are needed to decipher where rabbit lipocalins are located in VSNs.

### Sex-steroid receptors are modulated by the environment and may arise as a new type of vomeronasal organ pheromone receptors

Steroids are well-known for their essential role in animal physiology, particularly regarding the internal state of the individual. Less studied but also vital for animal behaviour is the role of steroids in chemosensation. These compounds are excreted by animals in urine or faeces, releasing a readout of its own biological state, and therefore acting as chemosignals/pheromones to be detected through the VNO of conspecifics (Doyle and Meeks, 2018). Specifically, oestrogens and oxytocin have proved to modulate VNO neural excitability inducing behavioural changes (Yano et al., 2015; Nakahara et al., 2020). VSNs are highly sensitive to excreted glucocorticoids, sex-steroids and bile acids that might act as pheromones (Doyle and Meeks, 2018). Indeed, sulphated compounds are the predominant VSNs ligands in female mice urine (Nodari et al., 2008). Therefore, sex-hormones play an important role in activating VSNs, modulating sensory signalling and contributing to the heterogeneity of VSNs response capabilities (Dey et al., 2015; Stowers and Liberles, 2016). However, the relationship between steroid cues and the receptors involved in its detection, as well as the neural pathway involved in behavioural response remain still unclear. Recently, it has been shown that V1R receptors are involved in the detection of sulphated oestrogens (Haga-Yamanaka et al., 2015) and bile acids (Wong et al., 2020). Therefore, V1R are likely to be the predominant receptors of steroids in mice, though V2Rs also seem to be involved (Doyle and Meeks, 2018). Noteworthily, sex-steroid receptors were found in VSNs, suggesting a potential role in signalling actions of pheromones (Ploβ et al., 2014). For instance, oestrogen receptor (ESR1) in the VNO is directly localised to neural progenitors and mediates neurogenesis during pregnancy in female mice (Oboti et al., 2015). In the rabbit VNO, we found progesterone and oestrogen receptors as well as other sex-steroid binding molecules, whose expression pattern is modulated by specific environmental conditions. This highlights the plastic capacity of sex-steroid receptors in the rabbit VNO, placing them as potential candidates to perceive particular external and internal cues and trigger specific signalling responses.

The complexity of steroid chemosensation suggests that there is not a universal rule governing sex-steroid pheromone vs. receptor activity (Doyle and Meeks, 2018). However, throughout evolution, receptors of different families may have acquired steroid sensitivity. We argue that sex-steroid and VR receptors in the VNO may be responsible for detecting sex-steroid cues and triggering signal transduction to brain centres. We propose two possible hypotheses: (1) sex-steroid receptors may act as a first screening of sensory perception in response to specific internal states followed by the activation of VRs of VSNs. Accordingly, we would expect VRs and sex-steroid receptors to be found in the same VSNs, to avoid energy loss due to cell-to-cell communication as well as to maintain high efficiency to trigger a quick response; and (2) both types of receptors, VRs and sex-steroid receptors perceive pheromones independently. In this case, we would expect VRs and sex-steroid receptors to be found in different VSNs and, accordingly, sex-steroid receptors could be considered as a new type of pheromone-receptors. Consequently, understanding the cellular rationale of the VNO will provide the needed framework for deciphering how pheromonal inputs are mediated by sensory neurons, potentially placing VSNs as a peripheral structure that integrates internal states with external chemosignals.

### A potential close relationship between the vomeronasal organ and female puberty

Females exposed to urine odours of males during pre-pubertal period accelerate puberty, a phenomenon known as Vandenbergh effect (Vandenbergh, 1967). Removal of either both olfactory bulbs or one olfactory bulb and sectioning the vomeronasal nerve abolished the rapid increase in uterine weight of immature female mice exposed to male mice, likely through pheromone-mediated behaviour (Kaneko et al., 1980). Sex-separated females that are exposed to male odours around the puberty period showed an increase in c-Fos activity (marker of neuronal activity) in the accessory olfactory bulb and higher centres of the vomeronasal pathway, arguing that such activity might be related to an earlier puberty onset (Szymanski and Keller, 2014). Additionally, it has been suggested that lesions of the VNO will block puberty acceleration in female mice exposed to male mouse urine (Szymanski and Keller, 2014). A recent study has determined that peripubertal VNO ablation decreased sexual odour preferences and neural activity in response to opposite-sex odours, suggesting that the VNO contributes to the sexual differentiation of behaviour and to the neural response to conspecific odours (Cross et al., 2021).

Our results indicate that the VNO expression pattern of juvenile females greatly differs between sex-separated and sex-combined conditions (SF vs. CF), whereas that of juvenile males remains almost unchanged. These results highlight the plasticity of the VNO in juvenile females exposed to different environmental conditions, which might be linked to female puberty. Regarding VRs, juvenile males and females showed similar VR gene expression repertoire when sex-combined (CM vs. CF). However, such VR repertoire vary in sex-separated conditions, showing overall upregulation in sex-separated females when compared to males and also when compared to their sex-combined counter partners.

We speculate that this VR upregulation in juvenile separated females might follow the theory “use it and lose it” (Xu et al., 2016; Van der Linden et al., 2018), suggesting that females not exposed to males would show standard VRs expression in a given VSN; however, after even short male odour stimulation, which is known to accelerate puberty, VRs expression would increase until a certain threshold, followed by selectively reduction of VRs expression and possibly in the abundance of specific VSN subtypes as well. In other words, pheromones contained in male urine would bind already activated VRs of separated females, and this would accelerate puberty onset; therefore, puberty onset would depend on the environmental condition and the sex-separated scenario would “prepare” VRs to receive pheromones from the opposite sex. Our data suggest that this interpretation would be only applicable for juveniles and puberty onset. VR gene expression proved to be extremely downregulated in separated adult females, which would therefore follow a complete different rule. One suggestion is the theory “use it or lose it” (Xu et al., 2016; Van der Linden et al., 2018). This means that females that are not exposed to males for a long period of time (6 months) might reduce their capacity to respond to male cues. Of note, downregulation of VRs in separated females contrasts with the upregulation of GPA1 in separated females and also in separated males, a G-protein known to mediate V1R and V2R signal transduction, raising the question of whether GPA1 could be implicated in other G protein coupled receptor pathways and therefore modulate VNO signalling in response to other vomeronasal receptors.

All in all, this study shows the plastic capacity of the rabbit VNO, and that in mammals this feature is not limited to mice. Our experimental design of sex-separation vs. sex-combined scenarios is a suitable model of environmental modulation to study VNO plasticity. We demonstrated that the rabbit VNO exhibits significant gene expression differences between male and female under sex-separation but not sex-combined scenarios. These differences are not only remarkable for the vomeronasal receptor repertoire but also for many genes involved in sensory perception, reproduction, immunity and VNO functional activity. Additionally, the low overlap between the different comparisons suggests that variation in the VNO gene expression under environmental conditions is both sex- and stage-specific.

Regulation of transcription arises as a mechanism that the VNO may use to adapt sensory responses to specific external stimuli. Further studies should approach the molecular logic of such transcriptional changes as well as its possible relationship with variation in the VNO cellular and neuron abundance.

## Data availability statement

The datasets presented in this study can be found in online repositories. The names of the repository/repositories and accession number(s) can be found below: https://www.ncbi.nlm.nih.gov/, PRJNA720622.

## Ethics statement

The animal study was reviewed and approved by Comité de Bioética da Universidade de Santiago de Compostela, Edificio CACTUS, CAMPUS DE LUGO, 27002 LUGO.

## Author contributions

PM, PS-Q, and PRV conceived and designed the experiments and RNAseq assay. PRV, JG, LQ, and PS-Q were involved in the experimental sampling. PRV and DR carried out the sequencing, transcriptomic analyses, bioinformatic work, and data analysis. PRV wrote the original draft. DR and PM participated in the reviewing and critical analysis of the manuscript. All authors provided critical input and approved the final version.

## Footnotes

1
https://www.bioinformatics.babraham.ac.uk/projects/fastqc/

2
https://www.ensembl.org/Multi/Tools/Blast

3
https://string-db.org/network/10090.ENSMUSP00000095655

## Abbreviations

APHR: aphrodisin
AQPs: aquaporins
Bpifb3: BPI fold containing family B member 3
CCK: cholecystokinin
CF: combined female
CM: combined male
DE: differentially expressed
DEGs: differentially expressed genes
ESR: oestrogen
FC: fold change
FDR: false discovery rate
FEL1A: major allergen I polypeptide chain 1
FLP: female-specific lacrimal gland protein
FPRs: formyl peptide receptors
GHRHR: growth hormone-releasing hormone receptor
GO: gene ontology
GPA1: alpha subunit of the heterotrimeric guanine nucleotide-binding G protein
H2-Mv: A multigene family of non-classical class I major histocompatibility complex (MHC) genes
LCN1_B: lipocalin 1B
LCN2: lipocalin 2
MHC: major histocompatibility complex
MSP: male-specific submandibular salivary gland proteins
MUP: major urinary protein
NCBI: National Center for Biotechnology Information
ORs: olfactory receptors
PCA: principal component analysis
PRG: progesterone
RNAseq: RNA sequencing
SF: separated female
SLC1A1: excitatory amino acid transporter 3
SM: separated male
SRA: short read archive
V1R: vomeronasal receptors type 1
V2R: vomeronasal receptors type 2
VNO: vomeronasal organ
VNS: vomeronasal system
VR: vomeronasal receptors
VSNs: vomeronasal sensory neurons.

## References

[B1] Ablimit A. Aoki T. Matsuzaki T. Suzuki T. Hagiwara H. Takami S. (2008). Immunolocalization of water channel aquaporins in the vomeronasal organ of the rat: Expression of AQP4 in neuronal sensory cells. *Chem. Senses* 33 481–488. 10.1093/chemse/bjn015 18407959

[B2] Alvaro C. G. Thorner J. (2016). Heterotrimeric G protein-coupled receptor signaling in yeast mating pheromone response. *J. Biol. Chem.* 291 7788–7795. 10.1074/jbc.R116.714980 26907689 PMC4824985

[B3] Berghard A. Buck L. B. (1996). Sensory transduction in vomeronasal neurons: Evidence for G alpha o, G alpha i2, and adenylyl cyclase II as major components of a pheromone signaling cascade. *J. Neurosci.* 16 909–918. 10.1523/JNEUROSCI.16-03-00909.1996 8558259 PMC6578816

[B4] Boillat M. Challet L. Rossier D. Kan C. Carleton A. Rodriguez I. (2015). The vomeronasal system mediates sick conspecific avoidance. *Curr. Biol.* 25 251–255. 10.1016/j.cub.2014.11.061 25578906

[B5] Bolger A. M. Lohse M. Usadel B. (2014). Trimmomatic: A flexible trimmer for Illumina sequence data. *Bioinformatics* 30 2114–2120. 10.1093/bioinformatics/btu170 24695404 PMC4103590

[B6] Brann J. H. Firestein S. (2010). Regeneration of new neurons is pre- served in aged vomeronasal epithelia. *J. Neurosci.* 30 15686–15694. 10.1523/JNEUROSCI.4316-10.2010 21084624 PMC3393108

[B7] Bray N. L. Pimentel H. Melsted P. Pachter L. (2016). Near-optimal probabilistic RNA-seq quantification. *Nat. Biotech.* 34 525–527. 10.1038/nbt.3519 27043002

[B8] Briand L. Blon F. Trotier D. Pernollet J. C. (2004a). Natural ligands of hamster aphrodisin. *Chem. Senses* 29 425–430. 10.1093/chemse/bjh044 15201209

[B9] Briand L. Trotier D. Pernollet J. C. (2004b). Aphrodisin, an aphrodisiac lipocalin secreted in hamster vaginal secretions. *Peptides* 25 1545–1552. 10.1016/j.peptides.2003.10.026 15374656

[B10] Broad K. D. Keverne E. B. (2012). The post-natal chemosensory environment induces epigenetic changes in vomeronasal receptor gene expression and a bias in olfactory preference. *Behav. Genet.* 42 461–471. 10.1007/s10519-011-9523-9 22179772

[B11] Bufe B. Schumann T. Zufall F. (2012). Formyl peptide receptors from immune and vomeronasal system exhibit distinct agonist properties. *J. Biol. Chem.* 287 33644–33655. 10.1074/jbc.M112.375774 22859307 PMC3460462

[B12] Bufe B. Teuchert Y. Schmid A. Pyrski M. Pérez-Gómez A. Eisenbeis J. (2019). Bacterial MgrB peptide activates chemoreceptor Fpr3 in mouse accessory olfactory system and drives avoidance behaviour. *Nat. Commun.* 10:4889. 10.1038/s41467-019-12842-x 31653840 PMC6814738

[B13] Carneiro M. Rubin C. J. Di Palma F. Albert F. W. Alföldi J. Martinez-Barria A. (2014). Rabbit genome analysis reveals a polygenic basis for phenotypic change during domestication. *Science* 345 1074–1079. 10.1126/science.1253714 25170157 PMC5421586

[B14] Chamero P. Leinders-Zufall T. Zufall F. (2012). From genes to social communication: Molecular sensing by the vomeronasal organ. *Trends Neurosci.* 35 597–606. 10.1016/j.tins.2012.04.011 22658923

[B15] Clancy A. N. Macrides F. Singer A. G. Agosta W. C. (1984). Male hamster copulatory responses to a high molecular weight fraction of vaginal discharge: Effects of vomeronasal organ removal. *Physiol. Behav.* 33 653–660. 10.1016/0031-9384(84)90386-x 6522485

[B16] Cross S. K. J. Martin Y. H. Salia S. Gamba I. Major C. A. Hassan S. (2021). Puberty is a critical period for vomeronasal organ mediation of socio-sexual behavior in mice. *Front. Behav. Neurosci.* 14:606788. 10.3389/fnbeh.2020.606788 33551763 PMC7862124

[B17] Dey S. Chamero P. Pru J. K. Chien M. S. Ibarra-Soria X. Spencer K. R. (2015). Cyclic regulation of sensory perception by a female hormone alters behaviour. *Cell* 161 1334–1344. 10.1016/j.cell.2015.04.052 26046438 PMC4501503

[B18] Dietschi Q. Tuberosa J. Rösingh L. Loichot G. Ruedi M. Carleton A. (2017). Evolution of immune chemoreceptors into sensors of the outside world. *Proc. Natl. Acad. Sci. U.S.A.* 114 7397–7402. 10.1073/pnas.1704009114 28652375 PMC5514743

[B19] Dobin A. Davis C. A. Schlesinger F. Drenkow J. Zaleski C. Jha S. (2013). STAR: Ultrafast universal RNA-seq aligner. *Bioinformatics* 29 15–21. 10.1093/bioinformatics/bts635 23104886 PMC3530905

[B20] Doyle W. I. Meeks J. P. (2018). Excreted steroids in vertebrate social communication. *J. Neurosci.* 38 3377–3387. 10.1523/JNEUROSCI.2488-17.2018 29519850 PMC5895034

[B21] Duyck K. DuTell V. Ma L. Paulson A. Yu C. R. (2017). Pronounced strain-specific chemosensory receptor gene expression in the mouse vomeronasal organ. *BMC Genom.* 18:965. 10.1186/s12864-017-4364-4 29233099 PMC5727874

[B22] Flores-Horgue L. Assens A. Fodoulian L. Marconi L. Tuberosa J. Haider A. (2022). Transcriptional adaptation of olfactory sensory neurons to GPCR identity and activity. *Nat. Commun.* 13:2929. 10.1038/s41467-022-30511-4 35614043 PMC9132991

[B23] Francia S. Pifferi S. Menini A. Tirindelli R. (2014). “Vomeronasal receptors and signal transduction in the vomeronasal organ of mammals,” in *Neurobiology of chemical communication*, ed. Mucignat-Caretta C. (Boca Raton, FL: CRC Press). 10.1201/b16511-11 24830038

[B24] Guillamón A. Segovia S. (1997). Sex differences in the vomeronasal system. *Brain Res. Bull.* 44 377–382. 10.1016/S0361-9230(97)00217-7 9370202

[B25] Haga-Yamanaka S. Ma L. Yu C. R. (2015). Tuning properties and dynamic range of type 1 vomeronasal receptors. *Front. Neurosci.* 9:244. 10.3389/fnins.2015.00244 26236183 PMC4501179

[B26] He J. Ma L. Kim S. Nakai J. Yu C. R. (2008). Encoding gender and individual information in the mouse vomeronasal organ. *Science* 320 535–538. 10.1126/science.1154476 18436787 PMC2602951

[B27] Herbison A. E. (2016). Control of puberty onset and fertility by gonadotropin-releasing hormone neurons. *Nat. Rev. Endocrinol.* 12 452–466. 10.1038/nrendo.2016.70 27199290

[B28] Ibarra-Soria X. Levitin M. O. Saraiva L. R. Logan D. W. (2014). The olfactory transcriptomes of mice. *PLoS Genet.* 10:e1004593. 10.1371/journal.pgen.1004593 25187969 PMC4154679

[B29] Ichikawa M. (1998). Neuronal development, differentiation, and plasticity in the mammalian vomeronasal system. *Zool. Sci.* 13 627–639. 10.2108/zsj.13.627

[B30] Ishii T. Hirota J. Mombaerts P. (2003). combinatorial coexpression of neural and immune multigene families in mouse vomeronasal sensory neurons. *Curr. Biol.* 13 394–400. 10.1016/s0960-9822(03)00092-7 12620187

[B31] Jang T. Singer A. G. O’Connell R. J. (2001). Induction of c-fos in hamster accessory olfactory bulbs by natural and cloned aphrodisin. *Neuroreport* 12 449–452. 10.1097/00001756-200103050-00006 11234744

[B32] Kaneko N. Debski E. A. Wilson M. C. Whitten W. K. (1980). Puberty acceleration in mice. II. Evidence that the Vomeronasal organ is a receptor for the primer pheromone in male mouse urine. *Biol. Reprod.* 22 873–878. 10.1095/biolreprod22.4.873 7397305

[B33] Kroner C. Breer H. Singer A. G. O’Connell R. J. (1996). Pheromone-induced second messenger signaling in the hamster vomeronasal organ. *Neuroreport* 7 2989–2992. 10.1097/00001756-199611250-00038 9116225

[B34] Lanuza E. Martin-Sánchez A. Marco-Manclús P. Cádiz-Moretti B. Fortes-Marco L. Hernández-Martínez A. (2014). Sex pheromones are not always attractive: Changes induced by learning and illness in mice. *Anim. Behav.* 97 265–272. 10.1016/j.anbehav.2014.08.011

[B35] Leinders-Zufall T. Ishii T. Chamero P. Hendrix P. Oboti L. Schmid A. (2014). A family of nonclassical class i mhc genes contributes to ultrasensitive chemodetection by mouse vomeronasal sensory neurons. *J. Neurosci.* 34 5121–5133. 10.1523/JNEUROSCI.0186-14.2014 24719092 PMC4050176

[B36] Liberles S. D. (2014). Mammalian pheromones. *Annu. Rev. Physiol.* 76 151–175. 10.1146/annurev-physiol-021113-170334 23988175 PMC4310675

[B37] Liberles S. D. Horowitz L. F. Kuang D. Contos J. J. Wilson K. L. Siltberg-Liberles J. (2009). Formyl peptide receptors are candidate chemosensory receptors in the vomeronasal organ. *Proc. Natl. Acad. Sci. U.S.A.* 106 9842–9847. 10.1073/pnas.0904464106 19497865 PMC2690606

[B38] Liu S. Y. Zhang C. J. Peng H. Y. Sun H. Lin K. Q. Huang X. Q. (2017). Strong association of SLC1A1 and DPF3 gene variants with idiopathic male infertility in Han Chinese. *Asian J. Androl.* 19 486–492. 10.4103/1008-682X.178850 27232852 PMC5507099

[B39] Love M. I. Huber W. Anders S. (2014). Moderated estimation of fold change and dispersion for RNA-seq data with DESeq2. *Genome Biol.* 15 550–550. 10.1186/s13059-014-0550-8 25516281 PMC4302049

[B40] Ma T. Gao H. Fang X. Yang H. (2011). Expression and function of aquaporins in peripheral nervous system. *Acta Pharmacol. Sin.* 32 711–715. 10.1038/aps.2011.63 21602841 PMC4009970

[B41] Mägert H. J. Cieslak A. Alkan O. Lüscher B. Kauffels W. Forssmann W. G. (1999). The golden hamster aphrodisin gene. Structure, expression in parotid glands of female animals, and comparison with a similar murine gene. *J. Biol. Chem.* 274 444–450. 10.1074/jbc.274.1.444 9867863

[B42] Marom K. Horesh N. Abu-Snieneh A. Dafni A. Paul R. Fleck D. (2019). The vomeronasal system can learn novel stimulus response pairings. *Cell Rep.* 27 676–684. 10.1016/j.celrep.2019.03.042 30995466

[B43] Martinez-Marcos A. Jia C. Quan W. Halpern M. (2005). Neurogenesis, migration, and apoptosis in the vomeronasal epithelium of adult mice. *J. Neurobiol.* 63 173–187. 10.1002/neu.20128 15729685

[B44] Messina A. Pulli K. Santini S. Acierno J. Känsäkoski J. Cassatella D. (2020). Neuron-derived neurotrophic factor is mutated in congenital hypogonadotropic hypogonadism. *Am. J. Hum. Genet.* 106 58–70. 10.1016/j.ajhg.2019.12.003 31883645 PMC7042563

[B45] Migeotte I. Communi D. Parmentier M. (2006). Formyl peptide receptors: A promiscuous subfamily of G protein-coupled receptors controlling immune responses. *Cytokine Growth Factor Rev.* 17 501–519. 10.1016/j.cytogfr.2006.09.009 17084101

[B46] Miyawaki A. Matsushita F. Ryo Y. Mikoshiba K. (1994). Possible pheromone-carrier function of two lipocalin. *EMBO J.* 13 5835–5842. 10.1002/j.1460-2075.1994.tb06927.x 7813422 PMC395557

[B47] Mohrhardt J. Nagel M. Fleck D. Ben-Shaul Y. Spehr M. (2018). signal detection and coding in the accessory olfactory system. *Chem. Senses* 43 667–695. 10.1093/chemse/bjy061 30256909 PMC6211456

[B48] Nakahara T. S. Camargo A. P. Magalhães P. H. M. Souza M. A. A. Ribeiro P. G. Martins-Netto P. H. (2020). Peripheral oxytocin injection modulates vomeronasal sensory activity and reduces pup-directed aggression in male mice. *Sci. Rep.* 10:19943. 10.1038/s41598-020-77061-7 33203885 PMC7673031

[B49] Nodari F. Hsu F. F. Fu X. Holekamp T. F. Kao L. F. Turk J. (2008). Sulfated steroids as natural ligands of mouse pheromone-sensing neurons. *J. Neurosci.* 28 6407–6418. 10.1523/JNEUROSCI.1425-08.2008 18562612 PMC2726112

[B50] Oboti L. Peretto P. (2014). How neurogenesis finds its place in a hardwired sensory system. *Front. Neurosci.* 8:102. 10.3389/fnins.2014.00102 24847202 PMC4023038

[B51] Oboti L. Ibarra-Soria X. Pérez-Gómez A. Schmid A. Pyrski M. Paschek N. (2015). Pregnancy and estrogen enhance neural progenitor-cell proliferation in the vomeronasal sensory epithelium. *BMC Biol.* 13:104. 10.1186/s12915-015-0211-8 26621367 PMC4665882

[B52] Oleari R. Massa V. Cariboni A. Lettieri A. (2021). The Differential roles for neurodevelopmental and neuroendocrine genes in shaping GnRH neuron physiology and deficiency. *Int. J. Mol. Sci.* 22:9425. 10.3390/ijms22179425 34502334 PMC8431607

[B53] Pelosi P. Knoll W. (2022). Odorant-binding proteins of mammals. *Biol. Rev. Camb. Philos. Soc.* 97 20–44. 10.1111/brv.12787 34480392

[B54] Ploβ V. M. Gebhart V. M. Gisder D. Wölz W. Jirikowski G. F. (2014). Localization of sex hormone binding globulin in the rat vomeronasal organ. *J. Chem. Neuroanat.* 61-62 120–123. 10.1016/j.jchemneu.2014.08.004 25154024

[B55] R Core Team (2017). *R: A language and environment for statistical computing.* Vienna: R Core Team.

[B56] Rivière S. Challet L. Fluegge D. Spehr M. Rodriguez I. (2009). Formyl peptide receptor-like proteins are a novel family of vomeronasal chemosensors. *Nature* 459 574–577. 10.1038/nature08029 19387439

[B57] Roberts S. A. Simpson D. M. Armstrong S. D. Davidson A. J. Robertson D. H. McLean L. (2010). Darcin: A male pheromone that stimulates female memory and sexual attraction to an individual male’s odour. *BMC Biol.* 8:75. 10.1186/1741-7007-8-75 20525243 PMC2890510

[B58] Santoro S. W. Jakob S. (2018). Gene expression profiling of the olfactory tissues of sex-separated and sex-combined female and male mice. *Sci. Data* 5:180260. 10.1038/sdata.2018.260 30512012 PMC6278690

[B59] Schaal B. Coureaud G. Langlois D. Giniès C. Sémon E. Perrier G. (2003). Chemical and behavioural characterization of the rabbit mammary pheromone. *Nature* 424 68–72. 10.1038/nature01739 12840760

[B60] Shugart Y. Y. Wang Y. Samuels J. F. Grados M. A. Greenberg B. D. Knowles J. A. (2009). A family-based association study of the glutamate transporter gene SLC1A1 in obsessive–compulsive disorder in 378 families. *Am. J. Med. Genet. B* 150B 886–892. 10.1002/ajmg.b.30914 19152386

[B61] Singer A. G. Macrides F. Clancy A. N. Agosta W. C. (1986). Purification and analysis of a proteinaceous aphrodisiac pheromone from hamster vaginal discharge. *J. Biol. Chem.* 261 13323–13326. 10.1016/S0021-9258(18)69307-X 3759967

[B62] Srikantan S. Parekh V. De P. K. (2005). CDNA cloning and regulation of twosex-hormone-repressed hamster tear lipocalins having homology withodorant/pheromone-binding proteins. *Biochim. Biophys. Acta* 1729 154–165. 10.1016/j.bbaexp.2005.04.008 15950295

[B63] Stopka P. Kuntová B. Klempt P. Havrdová L. Èerná M. Stopková R. (2016). On the saliva proteome of the Eastern European house mouse (*Mus musculus musculus*) focusing on sexual signalling and immunity. *Sci. Rep.* 6:32481. 10.1038/srep32481 27577013 PMC5006050

[B64] Stopkova R. Klempt P. Kuntova B. Stopka P. (2017). On the tear proteome of the house mouse (*Mus musculus musculus*) in relation to chemical signaling. *PeerJ* 5:e3541. 10.7717/peerj.3541 28698824 PMC5502090

[B65] Stowers L. Liberles S. D. (2016). State-dependent responses to sex pheromones in mouse. *Curr. Opin. Neurobiol.* 38 74–79. 10.1016/j.conb.2016.04.001 27093585 PMC4921285

[B66] Szymanski L. A. Keller M. (2014). Activation of the olfactory system in response to male odors in female prepubertal mice. *Behav. Brain Res.* 271 30–38. 10.1016/j.bbr.2014.05.051 24886778

[B67] Tian T. Liu Y. Yan H. You Q. Yi X. Du Z. (2017). AgriGO v2.0: A GO analysis toolkit for the agricultural community. *Nucleic Acids Res.* 45 W122–W129. 10.1093/nar/gkx382 28472432 PMC5793732

[B68] Tirindelli R. (2021). Coding of pheromones by vomeronasal receptors. *Cell Tissue Res.* 383 367–386. 10.1007/s00441-020-03376-6 33433690

[B69] Törönen P. Medlar A. Holm L. (2018). PANNZER2: A rapid functional annotation web server. *Nucleic Acids Res.* 46 W84–W88. 10.1093/nar/gky350 29741643 PMC6031051

[B70] Trouillet A. C. Moussu C. Poissenot K. Keller M. Birnbaumer L. Leinders-Zufall T. (2021). Sensory detection by the vomeronasal organ modulates experience-dependent social behaviors in female mice. *Front. Cell. Neurosci.* 15:638800. 10.3389/fncel.2021.638800 33679330 PMC7925392

[B71] Tsukahara T. Brann D. H. Pashkovski S. L. Guitchounts G. Bozza T. Datta S. R. (2021). A transcriptional rheostat couples past activity to future sensory responses. *Cell* 184 6326–6343.e32. 10.1016/j.cell.2021.11.022 34879231 PMC8758202

[B72] Van der Linden C. Jakob S. Gupta P. Dulac C. Santoro S. W. (2018). Sex separation induces differences in the olfactory sensory receptor repertoires of male and female mice. *Nat. Commun.* 9:5081. 10.1038/s41467-018-07120-1 30514924 PMC6279840

[B73] Vandenbergh J. G. (1967). Effect of the presence of a male on the sexual maturation of female mice. *Endocrinology* 81 345–349. 10.1210/endo-81-2-345 4952008

[B74] Villafranca-Faus M. Vila-Martín M. E. Esteve D. Merino E. Teruel-Sanchis A. Cervera-Ferri A. (2021). Integrating pheromonal and spatial information in the amygdalo-hippocampal network. *Nat. Commun.* 12:5286. 10.1038/s41467-021-25442-5 34489431 PMC8421364

[B75] Villamayor P. R. Cifuentes J. M. Fdz-de-Troconiz P. Sanchez-Quinteiro P. (2018). Morphological and immunohistochemical study of the rabbit vomeronasal organ. *J. Anat.* 233 814–827. 10.1111/joa.12884 30255591 PMC6231170

[B76] Villamayor P. R. Robledo D. Fernández C. Gullón J. Quintela L. Sánchez-Quinteiro P. (2021). Analysis of the vomeronasal organ transcriptome reveals variable gene expression depending on age and function in rabbits. *Genomics* 113 2240–2252. 10.1016/j.ygeno.2021.05.007 34015461

[B77] Weiß E. Kretschmer D. (2018). Formyl-peptide receptors in infection, inflammation, and cancer. *Trends Immunol.* 39 815–829. 10.1016/j.it.2018.08.005 30195466

[B78] Wong W. M. Cao J. Zhang X. Doyle W. I. Mercado L. L. Gautron L. (2020). Physiology-forward identification of bile acid–sensitive vomeronasal receptors. *Sci. Adv.* 6:eaaz6868. 10.1126/sciadv.aaz6868 32523992 PMC7259934

[B79] Xu P. S. Lee D. Holy T. E. (2016). Experience-Dependent plasticity drives individual differences in pheromone sensing neurons. *Neuron* 91 878–892. 10.1016/j.neuron.2016.07.034 27537487 PMC5003430

[B80] Yano S. Sakamoto K. Q. Habara Y. (2015). Female mice avoid male odor from the same strain via the vomeronasal system in an estrogen-dependent manner. *Chem. Senses* 40 641–648. 10.1093/chemse/bjv052 26377346

[B81] Yates A. D. Achuthan P. Akanni W. Allen J. Allen J. Alvarez-Jarreta J. (2020). Ensembl 2020. *Nuclei Acids Res.* 48 D682–D688. 10.1093/nar/gkz966 31691826 PMC7145704

[B82] Zhang X. Marcucci F. Firestein S. (2010). High-throughput microarray detection of vomeronasal receptor gene expression in rodents. *Front. Neurosci.* 4:164. 10.3389/fnins.2010.00164 21267422 PMC3024560

