# Supplementary material for: Sex separation unveils the functional plasticity of the vomeronasal organ in rabbits
Source: Front Mol Neurosci. 2022 Oct 21;15:1034254. doi: 10.3389/fnmol.2022.1034254 (PMC9634631; doi:10.3389/fnmol.2022.1034254)
Supplement: Supplementary file 1 [file Data_Sheet_1.pdf]

Supplementary Material

1 Supplementary Data

Supplementary Data 1: List of differential expressed genes between males and females for both sex-separated and sex-combined animals, considering juveniles and adults.

Supplementary Data 2: Overlapping genes considering DE female *vs* female

Supplementary Data 3: List of differential expressed genes between sex-separated and sex-combined individuals, for both males and females, considering juveniles and adults.

Supplementary Data 4: Overlapping genes considering DE sex-combined *vs* sex-separated

Supplementary Data 5: List of differential expressed vomeronasal receptor genes between males and females for both sex-separated and sex-combined animals, considering juveniles and adults

Supplementary Data 6: List of differential expressed vomeronasal receptor genes between sex-separated and sex-combined individuals, for both males and females, considering juveniles and adults.

Supplementary Data 7: Enrichment analysis of the DEGs among experimental comparisons (SF *vs* SM, CF *vs* CM, SF *vs* CF, SM *vs* CM; both in adults and juveniles – only when the number of DEGs was sufficient to perform the analysis)

Supplementary Data 8: List of genes belonging to the H2-Mv complex (vomeronasal receptors) in mice, and the corresponding genes found when blasted against the rabbit genome.

2 Supplementary Figures

Supplementary Figure 1

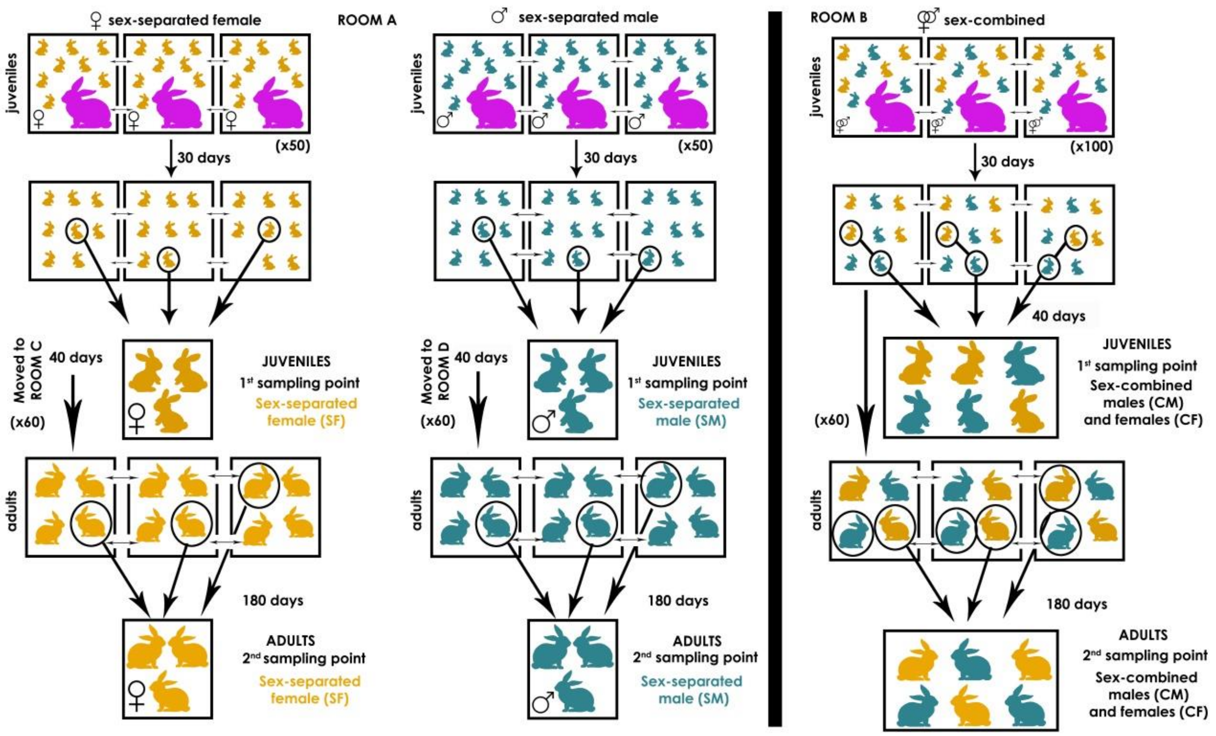

Supplementary Figure 1. Experimental design of sex-separation and sex-combined male and female, both for juvenile and adult individuals

Supplementary Figure 2

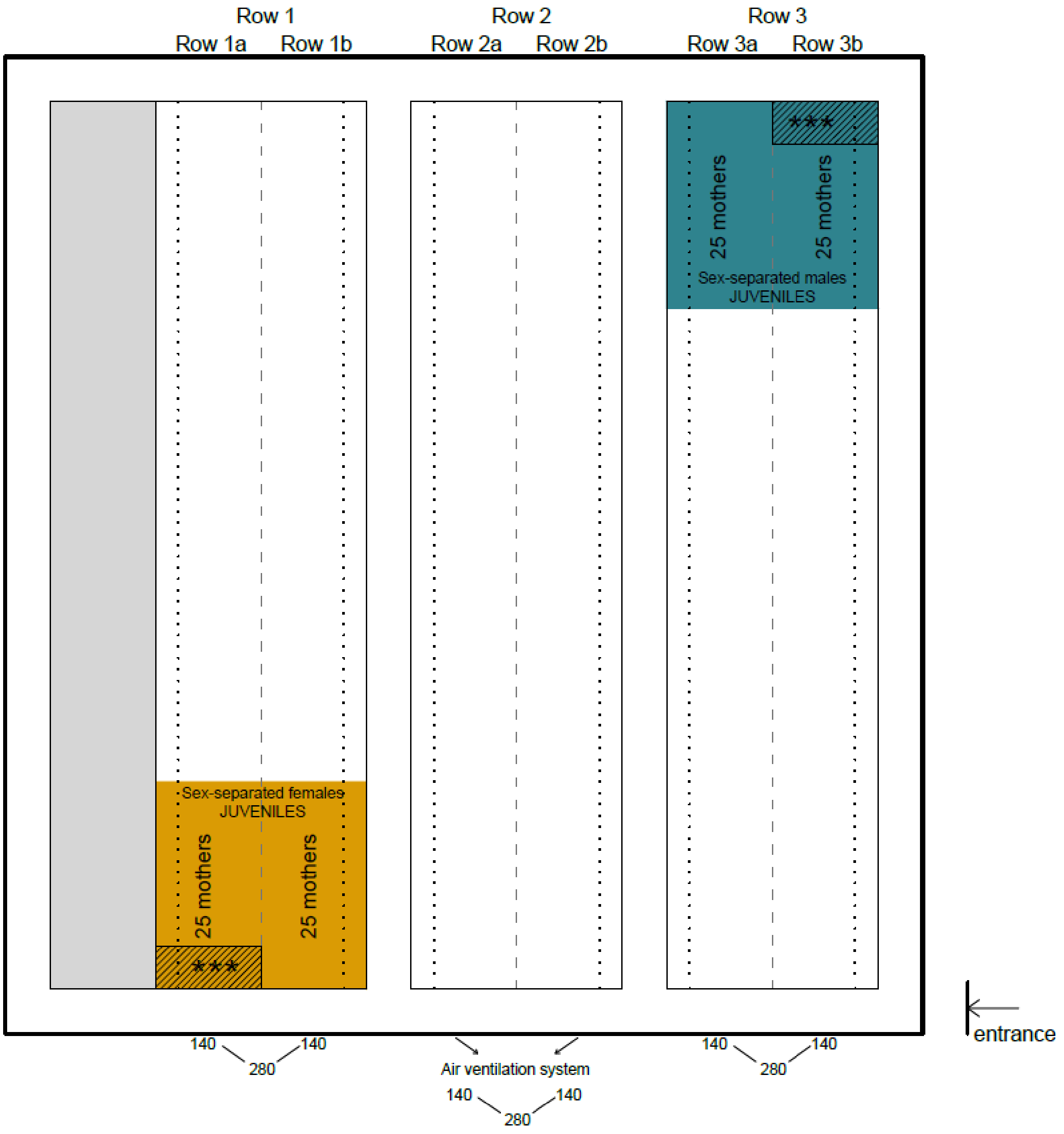

Supplementary Figure 2. Maps of the farm and experimental design organization. **Scheme of room A (sex-separated animals).** The room has three double rows (1a, 1b, 2a, 2b, 3a, 3b) were mothers and their litters are located and an additional row (in grey) is used as a backup to replace animals of the main chain when needed. Each double row contains 280 mothers, each with their own litter (8 kits/litter). Litters are always re-arranged at day 0 to assure that all kits/mother have similar size. Air ventilation system works from the side towards the center of the room and in one direction only (see

arrows in the scheme). Our experimental groups were of 50 mothers/experimental group and all eight kits/mother were either females or males for the sex-separated female and sex-separated male groups, respectively. The experimental groups (sex-separated female in orange and sex-separated male in green) were located according to the air ventilation system so that there is no air crossing. Weaning took place at day 30. Normal farm routing involves shifting of mothers to a different room to start the cycle again whereas the 8 kits / cage remain in the room until day 68, when they are sent to the slaughter house. In the experiment, mothers were taken out as usual farm routine, and the first **sampling point took place in room A at day 40,** in which 3 animals per group, each coming from a different mother, were used for the experiment. Animals were taken from the most distant sides of the room (indicated with ***). At day 40, 60 sex-separated males and 60 sex-separated females were shifted to rooms C and D respectively. The rest of the animals followed the normal farm routine.

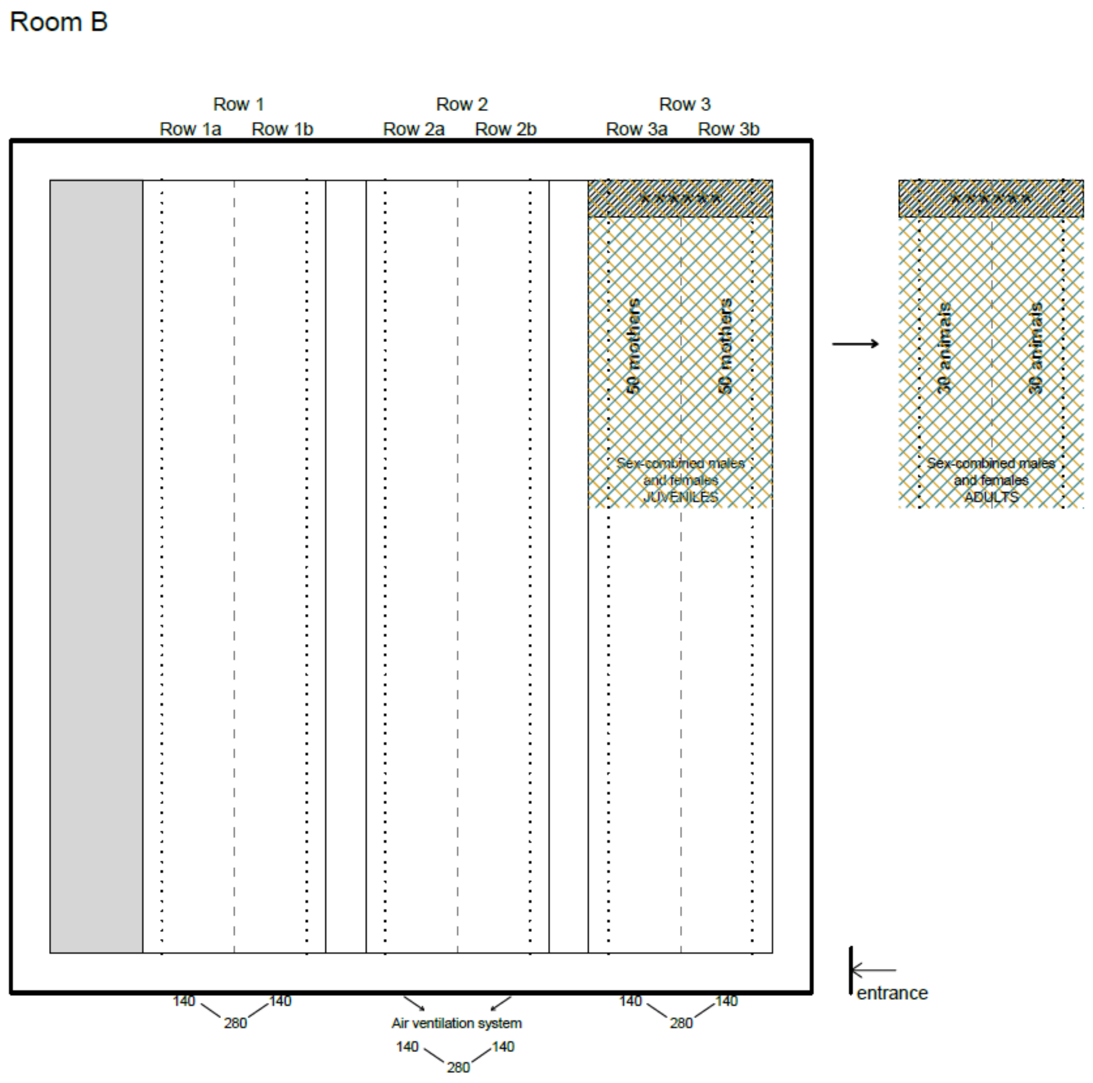

Scheme of room B (sex-combined animals). The room has the same features as room A. In this case, we employed 100 mothers, and each mother had 8 kits, 4 males and 4 females. Similarly to sex-separated condition, first sampling point took place at day 40, in which 6 animals (3 males and 3 females) (***), each coming from a different mother, were used for the experiment. Importantly, for the adults, animals were not shifted to another room; instead, they were kept in the same room to ensure common farm routine. Animals were re-arranged so that four individuals, two males and two females, were kept in each cage.

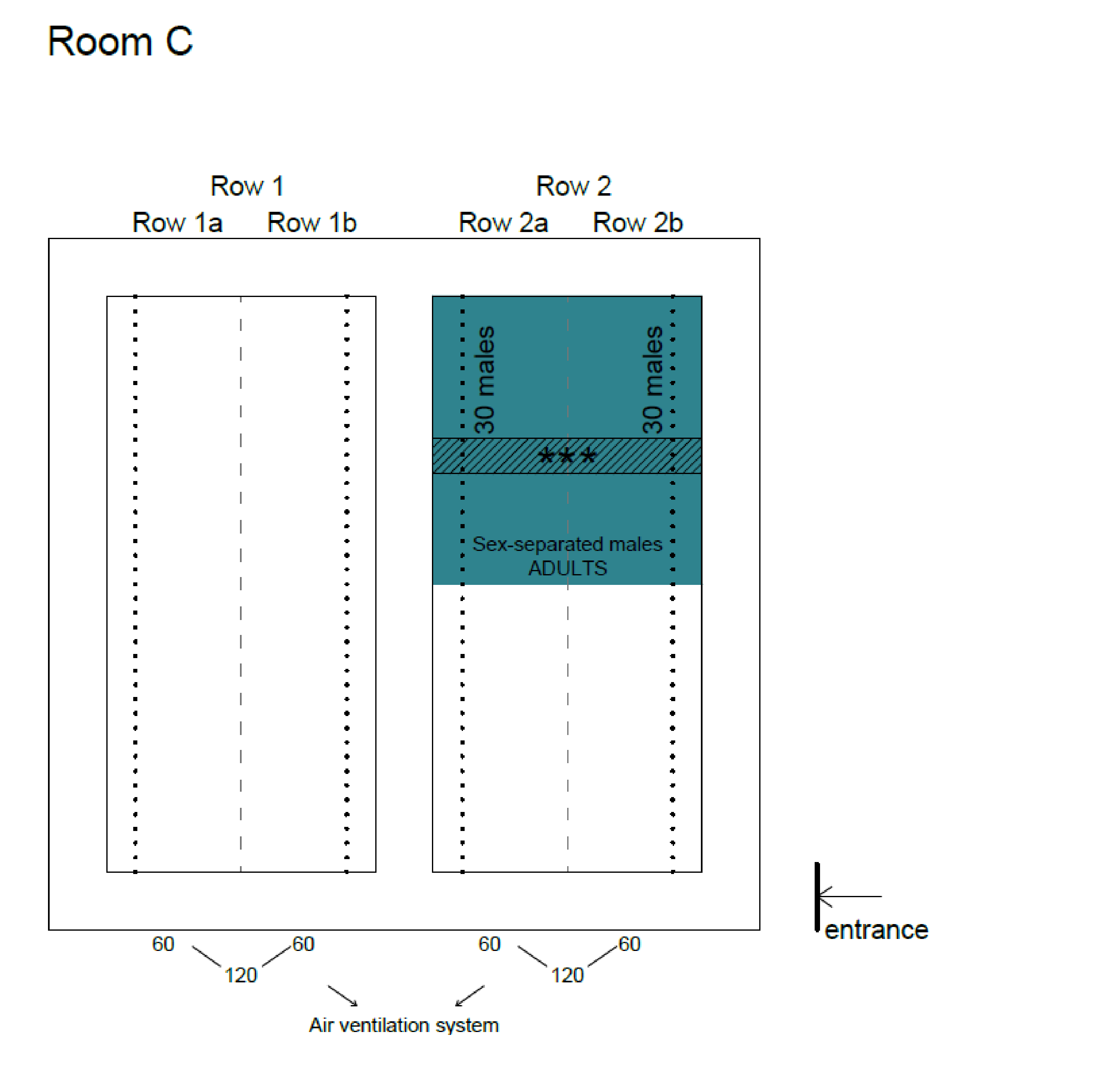

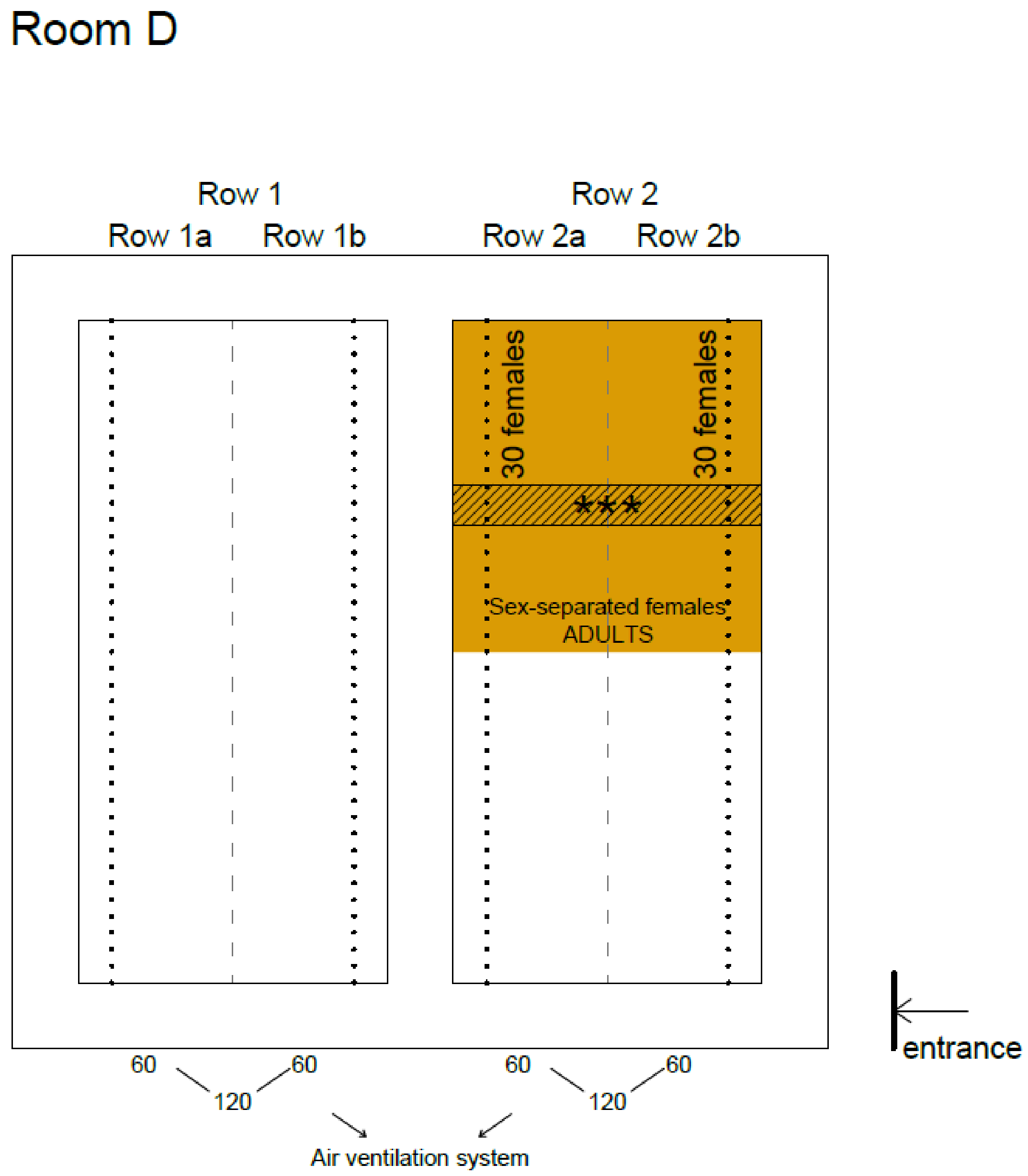

Scheme of room C, D (sex-separated males and females respectively). The room is considerably smaller than room A, containing three double rows (1a, 1b, 2a, 2b, 3a, 3b) and capacity for a total of 120 individuals. 60 sex-separated males and 60 sex-separated females were taken from room A at day 40 and placed in room C and room D, respectively. Four animals were placed / cage, assuring direct contact between individuals, and maintained until 6 months, in which three animals/experimental group from the most distant sides of the room (indicated with ***) were used for the experiment.

Additional information:

Some animals were eliminated due to health issues during the 6-month period. However, the outcome of the experiment was not affected thanks to the considerable high number of animals used to assure the 'sex-separation' and 'sex-combined' environments.

Supplementary Figure 3

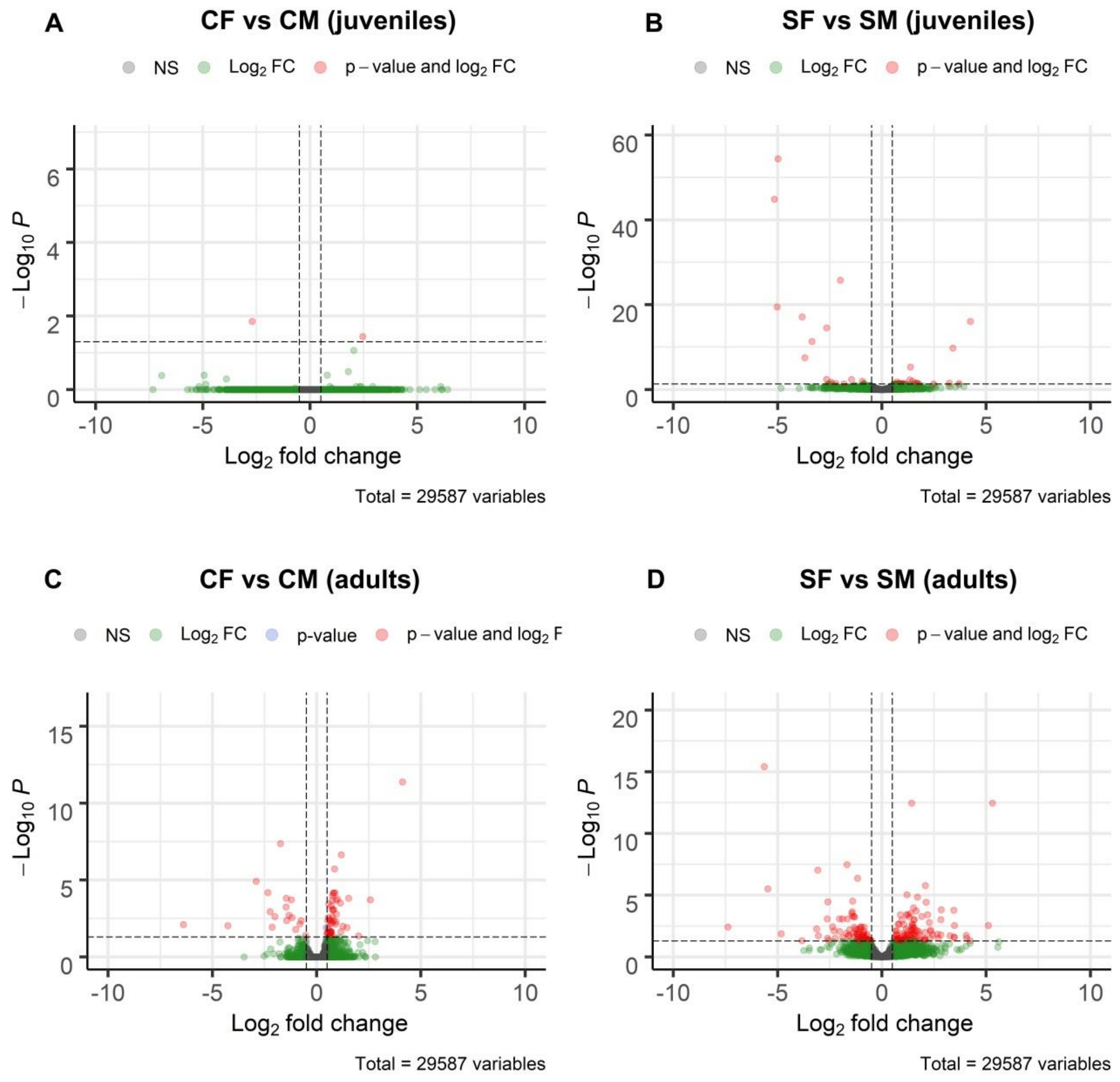

Supplementary Figure 3. Volcano plots showing differentially expressed genes (fold change (FC), abscissa) and its significance (-log P-value, ordinates) between female and male VNO RNAseq samples: **(a)** juveniles sex-combined **(b)** juveniles sex-separated **(c)** adults sex-combined and **(d)** adults sex-separated. Genes are classified in four categories depending on their FC and FDR corrected p-value: i) grey = p-value > 0.01 and log2 FC between -0.5 and 0.5; ii) green = p-value >

0.01 and log2 FC < -0.5 or > 0.5; iii) blue = p-value < 0.01 and log2 FC between -0.5 and 0.5; and iv) red = p-value < 0.01 and log2 FC < -0.5 or > 0.5). CF: sex-combined females; CM: sex-combined males; SF: sex-separated females; SM: sex-separated males.

Additional Figure 4

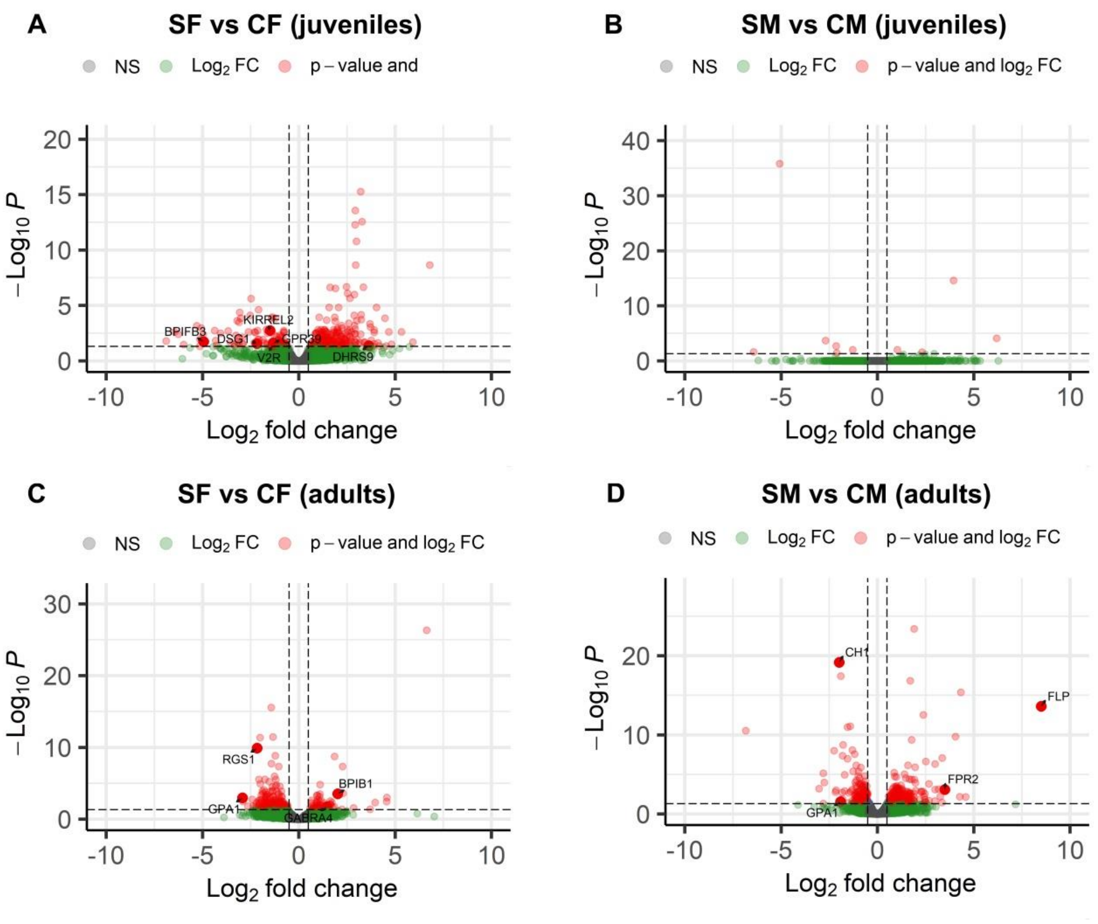

Supplementary Figure 4. Volcano plots showing differential expression between sex-separated and sex-combined VNO RNAseq samples for the comparisons studied: **(b)** juvenile females **(c)** juvenile males **(d)** adult females **(e)** adult males. Each point in the plot represents a gene, with its log2 fold change (FC) in the x-axis and its log10 p-value in the y-axis. Genes are classified in four categories depending on their FC and FDR corrected p-value: i) grey = p-value > 0.01 and log2 FC between -0.5 and 0.5; ii) green = p-value > 0.01 and log2 FC < -0.5 or > 0.5; iii) blue = p-value < 0.01 and log2 FC between -0.5 and 0.5; and iv) red = p-value < 0.01 and log2 FC < -0.5 or > 0.5).

Additional Figure 5

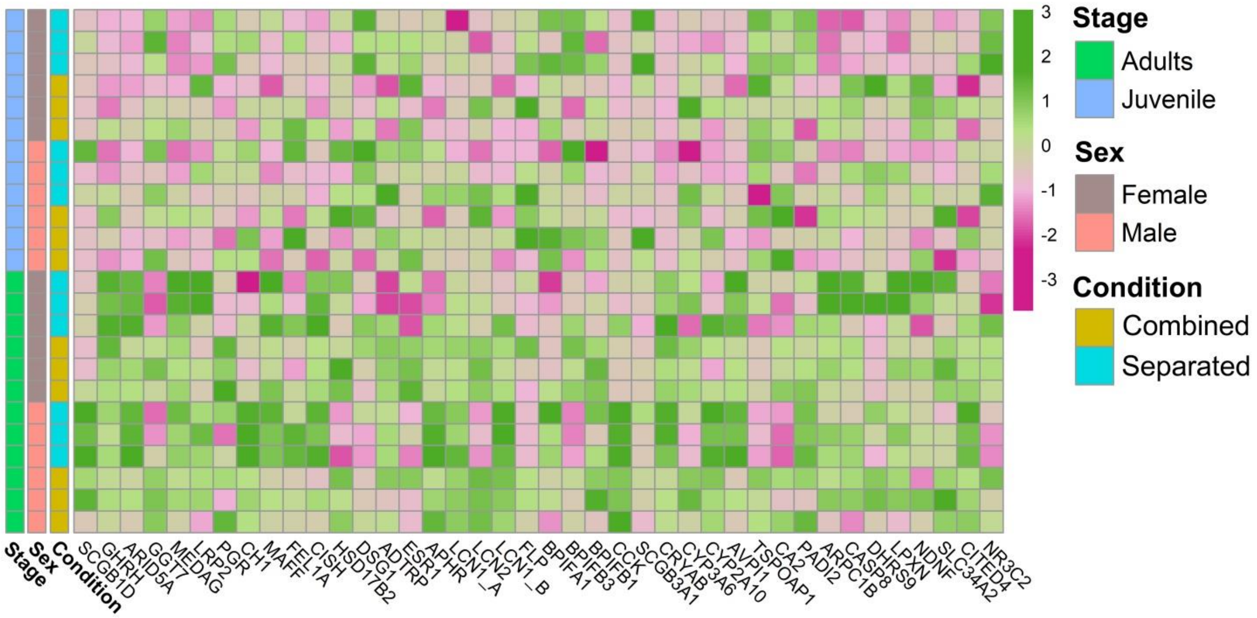

Supplementary Figure 5. Heatmap of reproductive-related genes differencially expressed between comparisons, both in juveniles and adults.

3 Supplementary Tables

Supplementary Table 1. Reproduction-related genes showing differential expression across socio-environmental conditions.

GENE NAME	Gene group	GENE FUNCTION
BPIFB3 (BPI Fold Containing Family B Member 3)	BPI family	BPI family. Interaction with lipocalins and major urinary proteins (MUPs). It may have the capacity to recognize and bind specific classes of odorants.
Lipocalins (LCN1_A, LCN1_B, LCN2)	Lipocalin	Bind putative sex-specific pheromones
APHR (aphrodisiac pheromone)	Lipocalin	Might bind putative pheromones
SCGB3A1 (Secretoglobin Family 3A Member 1)	Secretoglobin family	Secretoglobin family. Androgen binding
SCGB1D (Secretoglobin Family 1D Member 4)	Secretoglobin family	Secretoglobin family and lipophilin subfamily. Androgens and other steroid binding
Growth hormone-releasing hormone receptor (GHRHR),	Hormone receptor	Essential G-protein receptor in female reproduction
Progesterone receptor (PGR), estrogen receptor (ESR1)	Steroid binding	Steroid binding receptors, essential in female reproduction
CH1, FEL1A (Major allergen polypeptides)	Steroid binding	Steroid binding peptides
ARID5A (AT-Rich Interaction Domain 5A)	Steroid binding	Androgen/steroid binding
PADI2 (Protein-arginine deiminase type-2)	Steroid binding	Estrogen receptor binding
TSPOAP1 (Peripheral-type benzodiazepine receptor-associated protein 1), CA2 (Carbonic anhydrase 2), SLC34A2 (Sodium-dependent phosphate transport protein 2B)	Steroid binding	Steroid binding, respond to estrogens
NR3C2 (nuclear receptor subfamily 3 group C member 2)	Steroid binding	Steroid hormone receptor activity
NDNF (Protein NDNF)	Related to GnRH	Regulates GnRH neuronal migration to the hypothalamus (onset of puberty ([36] Herbison, 2016)
CISH (Cytokine Inducible SH2	Related to prolactin	Mediates prolactin inhibition

Containing Protein)		
MAFF (Transcription factor MafF)	Related to oxytocin	Interacts with the upstream promoter region of the oxytocin receptor gene
AVPI1 (arginine vasopressin induced 1)	Related to vasopressin	Vasopressin receptors have been found in the mice VNO (Nakahara et al. 2020)
DSG1 (Desmoglein-1)	Related to progesterone	Response to progesterone
CYP3A6 (Cytochrome P450 3A6)	Related to progesterone	Exhibits progesterone 6 beta-hydroxylase activity
CYP2A10 (Cytochrome P450 2A10)	Related to testosterone	Converts testosterone to androstenedione
HSD17B2 (17-beta-hydroxysteroid dehydrogenase type 2)	Related to testosterone and estrogens	Oxidizes estradiol to estrone, testosterone to androstenedione, and dihydrotestosterone to 5alpha-androstan-3,17-dione
MEDAG2 (Melanoma-associated antigen D2)	Related to estrogen	Mesenteric estrogen dependent adipogenesis. Involved in female pregnancy
CITED4 (Cbp/p300-interacting transactivator 4)	Related to estrogen	Enhances estrogen-dependent transactivation mediated by estrogen receptors; acts as a transcriptional coactivator for TFAP2/AP-2, previously implicated in vomeronasal function
CRYAB (Alpha-crystallin B chain), GGT7 (Glutathione hydrolase)	Related to estrogen	Responds to estradiol
SLC34A2 (solute carrier family 34 member 2)	Related to estrogen	Response to estrogen
ADTRP (Androgen dependent TFPI regulating protein)	Related to androgen	Androgen dependent protein. Cellular response to steroid hormone stimulus
LRP2 (Low-density lipoprotein receptor-related protein 2)	Related to androgen	Mediates uptake of androgen and estrogen in reproductive tissues
LPXN (Leupaxin)	Related to androgen	Transcriptional coactivator for androgen receptor
FLP (Female-specific lacrimal gland protein)	Lacrimal gland protein	Small molecule binding
CCK (Cholecystokinin)	Peptide hormone	Peptide hormone, regulated by estrogens
